# SAR Probing of KX2-391 Provided Analogues With Juxtaposed Activity Profile Against Major Oncogenic Kinases

**DOI:** 10.3389/fonc.2022.879457

**Published:** 2022-05-20

**Authors:** Abdelsattar M. Omar, Maan T. Khayat, Farid Ahmed, Yosra A. Muhammad, Azizah M. Malebari, Sara M. Ibrahim, Mohammad I. Khan, Dhaval K. Shah, Wayne E. Childers, Moustafa E. El-Araby

**Affiliations:** ^1^Faculty of Pharmacy, Department of Pharmaceutical Chemistry, King Abdulaziz University, Jeddah, Saudi Arabia; ^2^Centre for Artificial Intelligence in Precision Medicines, King Abdulaziz University, Jeddah, Saudi Arabia; ^3^Faculty of Pharmacy, Department of Pharmaceutical Chemistry, Al-Azhar University, Cairo, Egypt; ^4^Center of Excellence in Genomic Medicine Research, King Abdulaziz University, Jeddah, Saudi Arabia; ^5^Faculty of Applied Medical Sciences, King Abdulaziz University, Jeddah, Saudi Arabia; ^6^Faculty of Science, Department of Biochemistry, King Abdulaziz University, Jeddah, Saudi Arabia; ^7^School of Pharmacy and Pharmaceutical Sciences, Department of Pharmaceutical Sciences, University at Buffalo, State University of New York, Buffalo, NY, United States; ^8^Moulder Center for Drug Discovery Research, School of Pharmacy, Department of Pharmaceutical Sciences, Temple University, Philadelphia, PA, United States

**Keywords:** tirbanibulin, Src kinase, scaffold hopping, phosphokinase profiling, structure-properties relationship, leukemia, multi-kinase downregulation

## Abstract

Tirbanibulin (KX2-391, KX-01), a dual non-ATP (substrate site) Src kinase and tubulin-polymerization inhibitor, demonstrated a universal anti-cancer activity for variety of cancer types. The notion that KX2-391 is a highly selective Src kinase inhibitor have been challenged by recent reports on the activities of this drug against FLT3-ITD mutations in some leukemic cell lines. Therefore, we hypothesized that analogues of KX2-391 may inhibit oncogenic kinases other than Src. A set of 4-aroylaminophenyl-*N*-benzylacetamides were synthesized and found to be more active against leukemia cell lines compared to solid tumor cell lines. *N*-(4-(2-(benzylamino)-2-oxoethyl)phenyl)-4-chlorobenzamide (**4e**) exhibited activities at IC_50_ 0.96 µM, 1.62 µM, 1.90 µM and 4.23 µM against NB4, HL60, MV4-11 and K562 leukemia cell lines, respectively. We found that underlying mechanisms of **4e** did not include tubulin polymerization or Src inhibition. Such results interestingly suggested that scaffold-hopping of KX2-391 may change the two main underlying cytotoxic mechanisms (Src and tubulin). Kinase profiling using two methods revealed that **4e** significantly reduces the activities of some other potent oncogenic kinases like the MAPK member ERK1/2 (>99%) and it also greatly upregulates the pro-apoptotic c-Jun kinase (84%). This research also underscores the importance of thorough investigation of total kinase activities as part of the structure-activity relationship studies.

## 1 Introduction

Many drugs target oncogenic protein kinases by occupying the ATP site in the ternary complex, which is comprised of the apoenzyme, its specific substrate and an ATP molecule (1, 2). The most frequent issue of ATP-site kinase inhibitors is their off-target adverse effects due to structural similarities in the ATP binding sites (3). This lack of selectivity, however, can also be beneficial because small molecules that disrupt malignant tumor growth through inhibition of multiple targets are often powerful tools in cancer chemotherapy (4). Such multi-target monotherapies provide certain pharmacokinetic and pharmacodynamic (PK/PD) advantages over drug combinations (5–7). For instance, imatinib, the first FDA approved tyrosine kinase inhibitor (YKI), has been recognized as an inhibitor of BCR–ABL, c-KIT and PDGFR. Because of its multiple activities, imatinib has found use beyond the original designation for the treatment of chronic myeloid leukemia (CML) (8). Thus, polypharmacologic YKIs have become a common approach in this arena as researchers have become less concerned over the lack of kinase selectivity (9, 10).

In contrast, peptide site inhibitors have been a less popular strategy for the development of kinase inhibitors than the older ATP site inhibitors (11). After the marketing of the first ATP site inhibitor (imatinib), two decades passed before the approval of Tirbanibulin, the first-in-class substrate-site Src kinase inhibitor (12). Tirbanibulin (KX2-391) uniquely impedes mammalian cell division not only through Src inhibition, but also by binding to pretubulin leading to G2/M cell cycle arrest (13–15). Theoretically, peptide site inhibitors such as KX2-391, provide greater opportunities for kinase selectivity because of the characteristic amino acid sequence of their substrates (16). Nonetheless, this postulation has been recently challenged by a report of inhibition of FMS-Like Tyrosine Kinase 3-Internal Tandem Duplication (FLT3-ITD) in resistant acute myeloid leukemia (AML) cells (17). the proclaimed selectivity of non-ATP kinase inhibitors remains hypothetical unless the investigations include multiple kinase profiling of the inhibited cell line. Both sides of the debate about selectivity/polypharmacology have points of strength and weakness. Non-selective inhibition of multiple kinases may cause serios side effects. On the other hand, selective kinase inhibition may upregulate compensatory survival signaling pathways (18). The ultimate outcome (benefit vs. risk) should be the decisive parameter in evaluating compounds.

**Figure 1:**
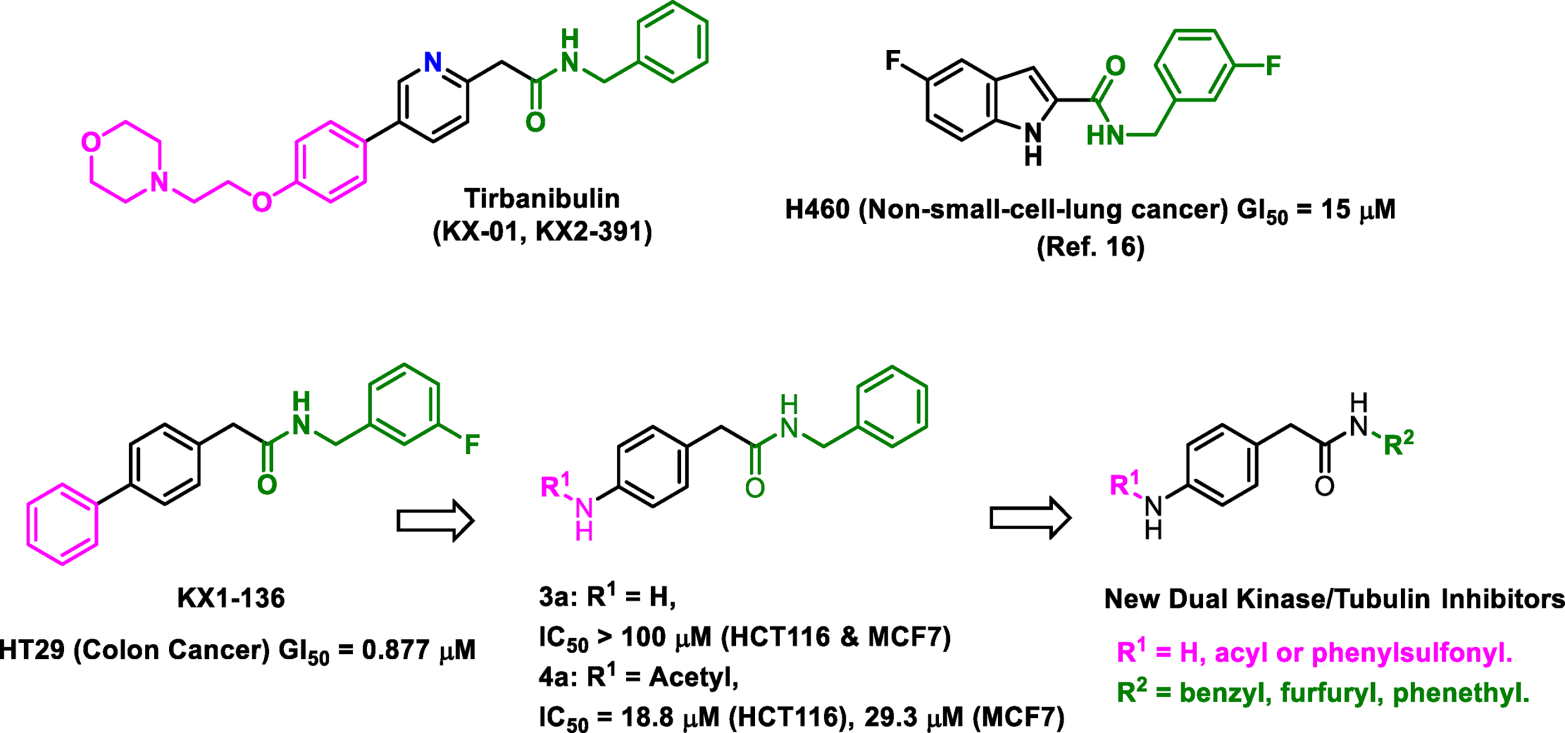
Structures of leading non-ATP competitive Src/tubulin dual inhibitors and the Scaffold-Hopping of KX1-136 into derivatives of 4-(acylamino)-*N*-(arylmethyl) phenylacetamide.

In this research, the medicinal properties of the 4-biphenyl-N-benzylacetamide pharmacophore (exemplified by KX1-136, **Figure 1**), a major lead for the development of KX2-391 (15), were modified by inserting an amide group between the two benzene rings of the biphenyl moiety and tested for cancer cell line cytotoxic activities. We hypothesized that the kinase activities of the new pharmacophore might possibly reveal either a kinase specificity shift or multiple kinase activities that disrupt oncogenic signaling pathways. One of the benefits of this change is to establish *N*-benzylarylacetamide as a privileged substructure to design kinase inhibitors regardless their selectivity.

## 2 Results

### 2.1 Rational Design

We sought to leverage the *N*-benzyl-4-biphenylacetamide scaffold as a prototypic chemical species for designing new antiproliferative agents. In this context, we hypothesized that the biaryl part of KX1-136 (GI_50_ against HT-29 colon cancer cell line = 0.877 µM) is not a strict requirement for antiproliferative activities (**Figure 1**). In fact, the Hangauer group reported Src inhibitors other than the biphenyl KX compounds, such as indole and naphthalene derivatives that showed moderate antiproliferative activities (**Figure 1**) (19–21). Therefore, we placed acylamino groups as convenient alternative to the outer phenyl ring with the intention of inhibiting cancer cell growth *via* intervention with oncogenic kinases regardless their relation to Src (**Figure 1**). The concept of employing amide groups in our scaffold-hopping process arose serendipitously when the intermediate 4-amino-*N*-benzylacetamide (**3a**) was unintentionally exposed to acetic anhydride to give the *N*-acetylated analogue **4a**. Upon screening, compound **4a** showed cytotoxic activities against colon (HCT116) and breast (MCF7) cancer cell lines, with IC_50_ values = 18.8 and 29.3 µM, respectively, while **3a** was devoid of any anticancer activities at concentrations up to 100 µM (**Figure 1** and **Table 1**). It was clear that the acetyl group conferred broad and reproducible anticancer activities, and this finding motivated us to investigate the SAR of the 4-acylaminophenyl-*N*-benzylacetamide derivatives (**Table 1**).

**Table 1:** Results of cytotoxic activities of compounds against variety of cancer cell lines expressed as IC_50_ ± SEM (µM).

Code	Solid tumor cell lines			Myelogenous leukemia cell lines		
	MCF7	HCT116	N87	MV4-11	K562	HL60
**3a**	>50	>50	>50	NT	NT	NT
**4a**	29.28 ± 4.64	18.83 ± 3.06	>50	NT	NT	NT
**4b**	27.70 ± 9.38	>50	32.02 ± 6.34	>20	>20	>20
**4c**	5.11 ± 3.03	19.69 ± 4.79	>50	11.87 ± 0.96	>20	5.27 ± 1.94
**4d**	6.55 ± 1.34	18.97 ± 4.18	>50	3.20 ± 0.09	2.68 ± 0.03	2.96 ± 0.25
**4e**	7.41 ± 2.80	16.75 ± 2.69	5.28 ± 0.91	1.90 ± 0.08	4.23 ± 0.20	1.62 ± 0.13
**4f**	>50	NT	48.33 ± 7.44	NT	NT	NT
**4g**	>50	>50	NT	>20	>20	>20

Note that we set the maximum tested concentration for solid tumor cell lines to 50 µM and the leukemia cell lines 20 µM.

### 2.2 Chemical Synthesis

**Scheme 1:**
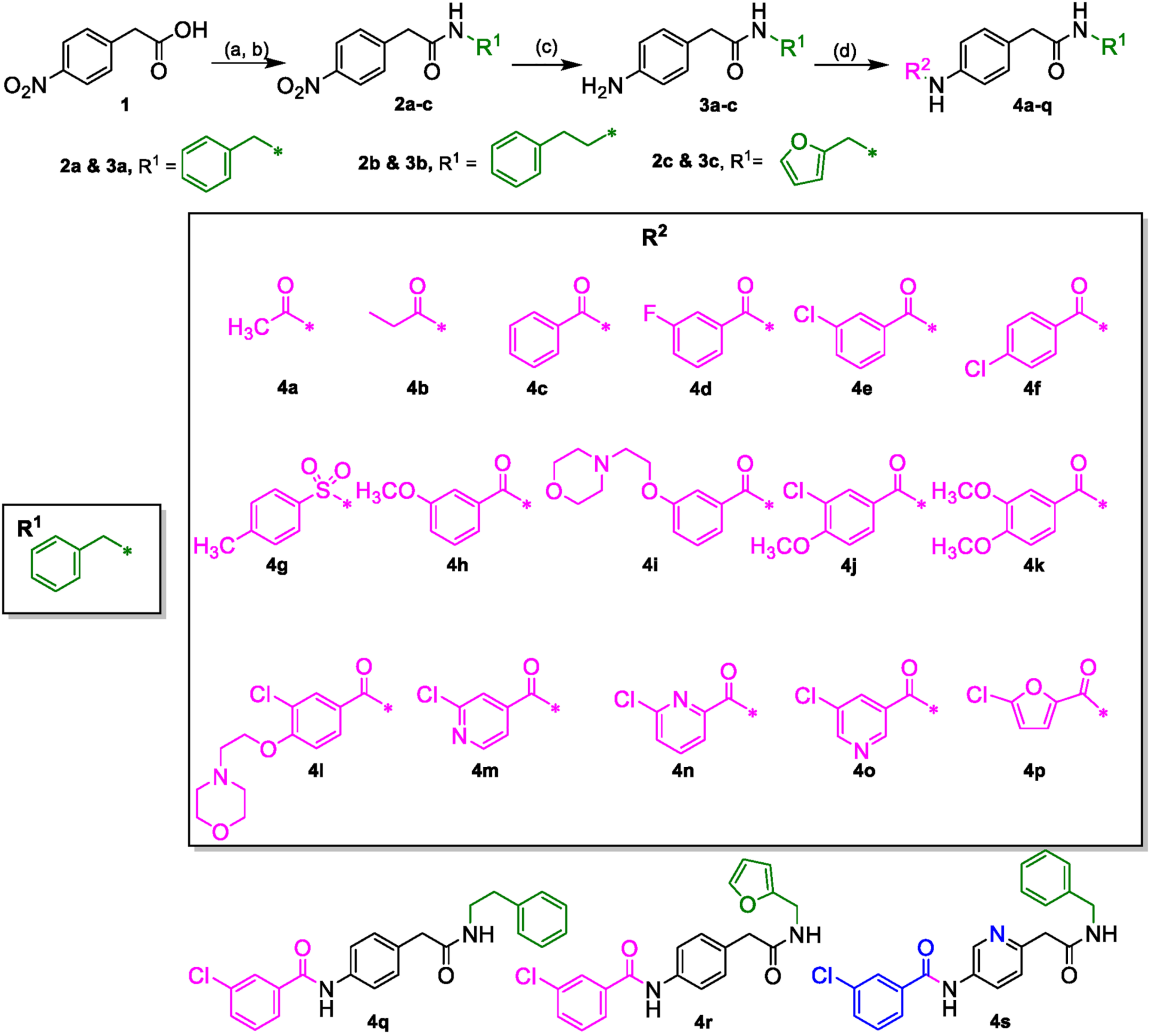
**(A)** (COCl)_2_, DCM, DMF (cat.). **(B)** R^1^NH_2_, DIPEA, DCM. **(C)** H-Cube Pro, Pd-C (10%), 10 atm, 40°C, ethyl acetate. **(D)** R^2^CO_2_H, EDCI·HCl, 3*H*-[1,2,3]triazolo [4,5-b]pyridin-3-ol, DMA or R^2^COCl, DIPEA, DCM.

The desired 4-acylamino-*N*-benzylphenylacetamide derivatives could be accessed from 4-nitrophenylacetic acid **1** in three steps *via* a straight-forward synthetic strategy. Compound 1 was converted to amides **2a-c *via*** a mixed anhydride procedure. Reduction of nitro group to afford amines **3a-c** was accomplished through hydrogenation, and acylation of **3a-c** provided the final products **4a**-**4s** (**Scheme 1**). Some acid intermediates were synthesized because they were not available from commercial sources such as those used to synthesize **4i** and **4l**. Descriptions of their synthesis can be found within the experimental section below. NMR and LC/MS spectra of the final target molecules can be found in the **Supplementary Material**.

### 2.3 Biological Screening

#### 2.3.1 Cancer Cell Line Viability Inhibition and Structure-Activity Relationships

One of the objectives of this research was to investigate the hypothesis that the new scaffold (4-aroyl-*N*-benzylarylacetamides) might engage other kinases than Src. Therefore, a variety of solid tumor and hematopoetic cancer cell lines was selected for screening regardless of their dependance on Src kinase, including stomach cancer (N87) and myelogenous leukemia (MV4-11, K562 and HL60) (22). Our results are presented in **Table 1**.

As a starting point for SAR, we observed that the unsubstituted aniline derivative **3a** showed no cytotoxicity against any of the cell lines at concentrations up to 50 μM. Since the early lead **4a** had a simple acetyl group, we decided to explore the nature of what appeared to be a defined pocket in a hypothetical binding site near this end of the scaffold. Therefore, a small set of compounds, **4b-g**, examining a variety of acyl groups was initially synthesized.

Compound **4b**, an aliphatic homologue of the aliphatic **4a**, demonstrated similar cytotoxic potency to that seen with **4a** against MCF7 cells. It lost the growth inhibition activity against HTC116 cells but showed some cytotoxic activity against N87 cells, an activity that **4a** lacked. Compound **4b** was also ineffective at preventing growth in the myelogenous leukemia cell lines. The benzoyl derivative **4c** was more potent than **4b** at inhibiting cell growth in MCF7 cells (IC_50_ = 5.11 µM) and demonstrated cytotoxic activity against MV4-11 and HL60 leukemia cells (IC_50_ = 11.87 and 5.27 μM, respectively). Therefore, we elected to investigate more derivatives of the aromatic **4c** and discontinued our efforts on the less promising aliphatic amides.

The *meta*-fluoro (**4d**) and *meta-*chloro (**4e**) analogues of **4c** exhibited comparable inhibition of MCF7 cell growth (IC_50_ = 6.55 and 7.41 µM, respectively) and also demonstrated cytotoxicity against K562 cells. In particular, **4e** was 10-fold more potent than the unsubstituted **4c** against the MV4-11 cell line, and also picked up cytotoxic activity against the stomach cancer cell line N87 (IC_50_ = 5.28 µM). The *para*-chloro substitution pattern (**4f**) was significantly less potent at inhibiting growth in all six cell lines, suggesting a size limitation in this position. The sulfonamide derivative **4g** was devoid of cytotoxic activity in our screens, but it was not clear if this was caused by the isosteric replacement of the amide or by the presence of a *para*-methyl group. Since our data indicated that the *meta*-substituted benzamides **4d** and **4e** (and especially the *meta*-chloro analog **4e**) were effective in all of the cell lines tested (single digit micromolar IC_50_ values except in HTC116 cells), we decided to expand upon our findings by synthesizing additional *meta*-substituted analogs with an emphasis on anti-proliferative activity in myelogenous leukemia by adding the acute promyelocytic leukemia cell line NB4 to the screening scheme. Those data are presented in **Table 2**.

**Table 2:** Results of cytotoxic activities of compounds against leukemia cell lines expressed as IC_50_ ± SEM (µM). MV4-11, HL60 and NB4 are AML cell lines. K562 cell line is CML cell line.

Code	MV4-11	HL60	NB4	K562
**4h**	2.40 ± 0.49	3.27 ± 0.15	1.96 ± 1.33	4.23 ± 2.44
**4i**	16.26 ± 4.43	>20	>20	17.36 ± 2.63
**4j**	20.00 ± 0.16	>20	5.33 ± 1.43	13.04 ± 0.04
**4k**	7.950 ± 0.31	>20	9.88 ± 2.80	>20
**4l**	18.85 ± 0.12	>20	8.77 ± 1.75	12.96 ± 0.08
**4m**	6.33 ± 4.24	>20	3.46 ± 0.06	12.44 ± 1.39
**4n**	1.96 ± 0.47	2.22 ± 0.06	1.54 ± 0.66	2.69 ± 0.65
**4o**	>20	>20	5.89 ± 1.11	>20
**4p**	>20	>20	>20	>20
**4q**	>20	>20	NT	>20
**4r**	>20	>20	NT	>20
**4s**	2.87 ± 0.3	6.24 ± 3.3	1.74 ± 0.16	4.00 ± 1.5

We explored more options at the *meta* position of the outer benzene ring by replacing the chloro group of the new lead **4e** with methoxy (**4h**) or the aqueous solubility promoting 2-morpholinoethoxy group (**4i**). Compound **4h** showed good broad spectrum antiproliferative activity that was comparable to that of **4e** with the exception that cytotoxic activity in the HL60 cell line was lost. Unfortunately, the bulkier **4i** was extremely weak. Disubstituted benzoyl analogues such as **4j**, **4k** and **4l** also showed weaker activities compared to **4e**, confirming the detrimental consequences of *para* substitution. The *ortho-*substituted analogues will be reported in a future publication because their activities and underlying mechanisms are very different from **4a**-**4s**.

**Figure 2:**
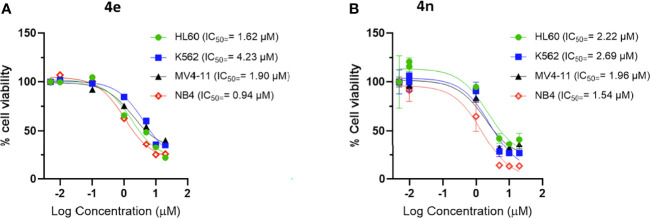
Dose response curve of **(A)** **4e** and **(B)** **4n** on leukemia cell lines. Cells were incubated with increasing concentration of compounds for 48 **h** and viability was assessed using CellTiter**^®^**-Blue Cell Viability Assay kit.

Aza-congeners of **4e** (**4m-4o** and **4s**) were then explored. These pyridyl derivatives provided antileukemic activities that depended on the position of the inserted ring nitrogen. The internal-ring pyridyl analogue **4s** showed comparable activities to **4e** with IC_50_ values 2.87, 6.24, 1.74 and 4.0 µM against MV4-11, HL60, NB4 and K562 cell lines, respectively. The 6-chloro-2-pyridyl analogue **4n** demonstrated good cytotoxic activities against the four tested leukemia cell lines MV4-11, HL60, NB4 and K562 with IC_50_’s at 1.96, 2.22, 1.54 and 2.69 µM, respectively (also comparable to that of **4e**) (**Figure 2**). Other pyridyl analogues **4m** and **4o** were not as potent as the phenyl analogue **4e**, especially **4o**, which was not active against MV4-11, HL60 and K562 cells at concentrations up to 20 μM. Replacing the left-hand phenyl group of **4e** with the electron rich 2-furanyl moiety (**4p**) also caused a sharp decline in anti-proliferative activity in all four leukemia cell lines (IC_50_ >20 µM).

Finally, to explore the SAR of the right-hand aryl ring we synthesized two analogues **4q** (*N*-phenethyl) and **4r** (*N*-(2-furylmethyl)) which probed spatial and electronic changes in *N*-benzyl group R^1^. Neither analogue inhibited the growth of the tested leukemia cell lines at concentrations up to 20 μM.

Clearly, compounds **4e**, **4h**, **4n** and **4s** showed similar levels of anti-leukemic activity on MV4-11, HL60 and K562 cell lines, although **4n** appeared slightly more potent than the other three derivatives. Since the NB4 cell line had not been included in the original screening round, we examined the effect of **4e** in this assay. Compound **4e** gave an excellent IC_50_ value (0.94 µM) against NB4, which was slightly better than that of **4h** (1.96 µM), **4n** (1.54 µM) and **4s** (1.74 µM). In addition, we tested **4n** for its inhibition of solid tumor cell lines and it exerted high potency against MCF7 (IC_50_ = 0.25 µM) and weaker activities against HCT116 (IC_50 =_ 10.76 µM). Based on their potency and broad spectrum anti-proliferative activity, we selected the m-chlorophenyl analogue **4e** and its aza analogue **4n** for further profiling and mechanism of action (MoA) studies.

#### 2.3.2 Profiling of Compounds 4e and 4n

There is a universal agreement that the physicochemical and pharmacokinetic properties play an important role in the viability of compounds as leads that can be optimized into drug candidates. Early *in vitro* screening of compounds in high throughput assays that predict *in vivo* ADME properties has become the norm for drug discovery, both in the industrial and academic setting (22). Properties such as aqueous solubility and protein binding impact not only *in vivo* studies but also biochemical and *in vitro* cellular assays as well. Cancer cells are well known to express elevated levels of hydrolytic enzymes as well, so assessment of the potential liability to hydrolytic metabolism is another important consideration for anti-cancer hits and leads especially that our compounds contain two amidic functionalities.

In addition to having a good physicochemical profile, it is important for a studied lead to differentiate between cancer cells and highly proliferating non-cancerous cells. Therefore, we assessed compounds **4e** and **4n** for both the *in silico* and *in vitro* physicochemical characteristics as well as for cytotoxic effect on human skin fibroblasts (HSF) cell lines.

##### 2.3.2.1 Calculated Physico-Chemical Properties

We employed the Canvas™ application provided in the Schrödinger Suite (Schrodinger, New York, New York) to assess the compliance of compounds **4e** and **4n** with Lipinski’s Rule of Five (Ro5) and Veber’s parameters (rotatable bonds and polar surface area) (23). The results are shown in **Table 3**.

**Table 3:** Calculated physico-chemical properties of the most active compounds, **4e** and **4n**.

Comp. No.	MW	Alog*P*	HBA	HBD	RB	PSA
**4e**	378.8	4.10	2	2	6	58.2
**4n**	379.8	3.59	3	2	6	71.1

The calculations revealed that the two compounds have no obvious violations of Lipinski’s or Veber’s rules (24, 25). However, we remained concerned about the aqueous solubility of the compounds since they contained carboxamides, which are known to reduce aqueous solubility, and did not contain any ionizable groups that could be used to prepare water-soluble salts. The compounds also contain a chloro group which is usually unfavorable to aqueous solubility (26). In addition, the amide groups might be susceptible to hydrolysis by cardiovascular amidases and other hydrolytic (non-redox) liver enzymes. This is an important issue that needed to be addressed to avoid overestimating the druggability of **4e** and **4n**.

##### 2.3.2.2 Solubility, Microsomal Stability, and Plasma Protein Binding

To address our concerns we tested compounds **4e** and **4n** in a battery of *in vitro* ADME assays, which included maximum kinetic aqueous solubility in 2% DMSO/phosphate-buffered saline (PBS), stability in mouse and human liver microsomes in the presence and absence of nicotinamide adenine di-nucleotide phosphate (NADPH), inhibitory activity against the three major human cytochrome P450 metabolizing enzymes (CYP3A4, CYP2D6 and CYP2C9), stability in mouse plasma at 37°C and plasma protein binding (expressed as percent bound to mouse plasma protein at 37°C). The results are summarized in **Table 4**.

**Table 4:** *In Vitro* Physicochemical/ADME Data and human skin fibroblasts (normal cell line) cytotoxicity for **4e** and **4n**.

Comp. No.	Max. aq. Sol. (µM)	Stability MLM t_1/2_, min	Stability HLM t_1/2_ (min)	Mic. Ctrl. Mouse% 60 min*	Mic. Ctrl. Human% 60 min*	CYP3A4 IC_50_ (µM)	CYP2D6 IC_50_ (µM)	CYP2C9 IC_50_ (µM)	Mouse Plasma Stability t_1/2_ (min)	Mouse PPB (% free)	HSF IC_50_ (µM)
**4e**	<2.0	>60	>60	73	102	>10	>10	>10	>300	0.8	>20
**4n**	2.6	3.1	40.1	5.0	98	>10	>10	>10	178	Unstable	0.27 ± 0.03

*No NADPH added.

As expected, compounds **4e** and **4n** displayed low to moderate solubility in 2% DMSO/PBS. Compound **4e** was extremely stable to oxidative metabolism by mouse and human liver microsomes in the presence of NADPH. There was a non-significant loss of compound **4e** in mouse liver microsomes lacking NADPH, a trend that was not repeated in human liver microsomes lacking NADPH. Compound **4e** was also stable in mouse plasma at 37°C for the duration of the assay (5 hours). These data suggest that **4e** is relatively stable to enzymatic hydrolysis in liver and cardiovascular tissue. Compound **4e** displayed high binding to mouse plasma proteins, with 0.8% free, unbound fraction detected. It showed no inhibition of the three major metabolizing CYP450 enzymes at concentrations up to 10 mM.

The aza variant **4n** was only marginally more soluble than **4e** in 2% DMSO/PBS (maximum solubility = 2.6 µM). However, this analogue was more labile in liver microsomes in the absence of NADPH than **4e**, especially in mouse liver microsomes. These data suggest that **4n** might be labile to enzymatic hydrolysis in liver tissue, an observation that was supported by the analog’s reduced stability in mouse plasma compared to that seen with **4e**. Compound **4n** also did not display any inhibitory activity against metabolizing CYP450 enzymes, but its reduced stability in mouse plasma made testing its protein binding in mouse plasma impossible.

Due to the modest aqueous solubility, we monitored carefully the solubilization of **4e** in assay conditions and kept maximum concentration in tests at 20 µM. All biological tests were checked for precipitation of the compound.

##### 2.3.2.3 Normal Cell Line Assay

The effect of the two compounds was evaluated for their effect on the growth and proliferation human skin fibroblasts (HSF) as an example of non-cancerous cell lines. Compound **4e** did not cause any significant inhibition of these cells (IC_50_ >20 µM) while the **4n** had potent cytotoxic activities in this assay.

Based on the above profiling results, we decided to focus on **4e** for cellular mechanism of action (MoA) studies to help us understand how these compounds elicited selective cytotoxicity in cancer cells.

#### 2.3.3 Effect of 4e on the Cell Cycle Distribution of HL60 Cell Line

**Figure 3:**
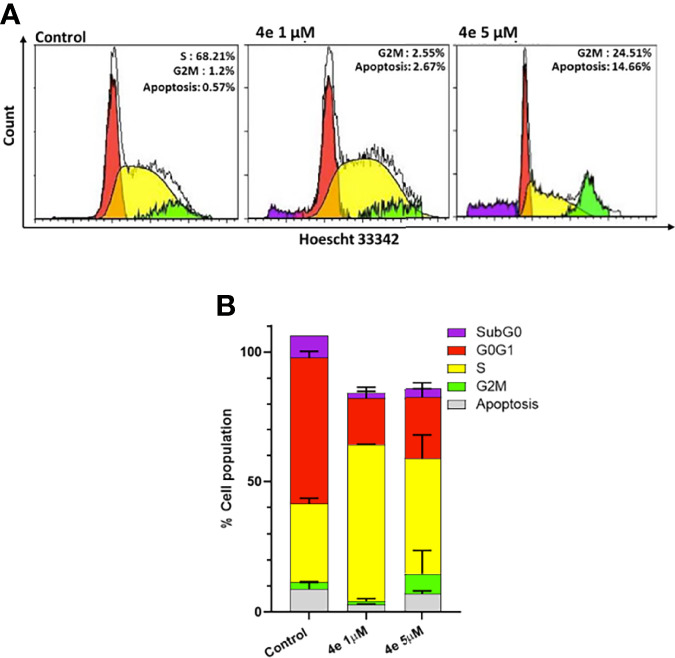
Cell cycle profile of **4e** in HL60 cell line. **(A)** Cells were treated with **4e** (1 µM and 5 µM) for 48 **h** and stained with Hoescht 33342 for analysis by flow cytometry. **(B)** Representative bar graph of the percentage of cells in different stages of cell cycle as mean ± SEM of two independent experiments.

The effect of **4e** on the distribution of cells in different phases of the cell cycle was determined by flow cytometry using Hoescht 33342 staining in HL60 cells. As shown in **Figure 3A**, **4e** increased the population of cells in the G2M phase at 1 µM concentration while also slightly increasing the percentage of apoptotic cells. Considerable increase in G2M population (24.51%) and apoptosis (14.66%) was observed at by **4e** at 5 µM in HL60 cell line as compared to the untreated control. Thus, **4e** induces apoptosis and blocks cell cycle in the G2M phase (**Figure 3B**).

#### 2.3.4 Induction of Apoptosis by 4e in HL60 Cells

**Figure 4:**
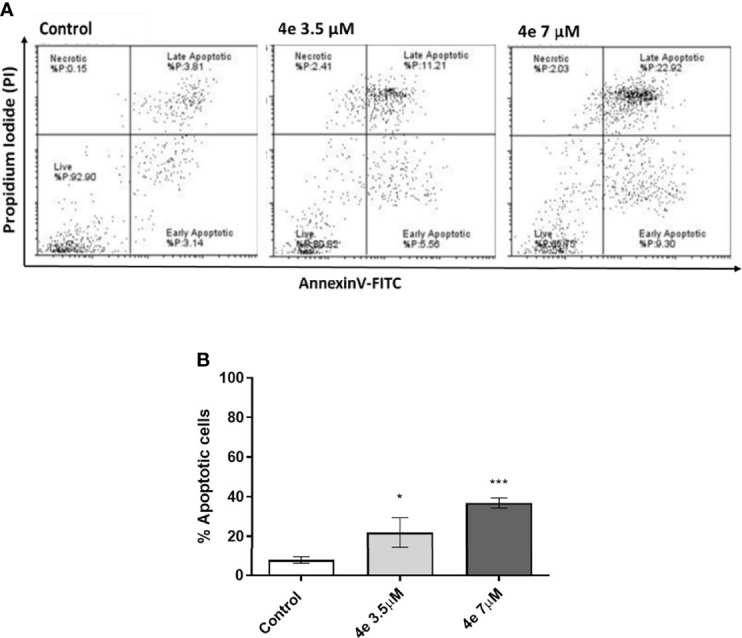
Total apoptosis produced by **4e** in HL60 cell line. **(A)** Apoptosis was measured *via* flow cytometry by AnnexinV-FITC/PI double staining upon 48 h treatment of cells with different concentrations of **4e**. **(B)** Representative bar graph of total percentage of apoptotic cells as mean ± SEM of three independent experiments. ^*^p < 0.05; ^***^p < 0.0005 as compared to the control.

Integral to the cell cycle analysis of **4e**, we determined the population of cells undergoing apoptosis, or programmed cell death, by Annexin V-FITC/PI double staining in HL60 cells. Two different concentrations of **4e** (3.5 µM and 7 µM) were selected for treatment. As shown in **Figure 4A**, treatment of HL60 cells with 3.5 µM **4e** moderately increased the percentage of apoptotic cells to 25.85%, which increased significantly at the higher dose (7 µM). Thus, **4e** dose dependently increased apoptosis in the HL60 cell line (**Figure 4B**).

#### 2.3.5 Effect of 4e on Tubulin Polymerization

**Figure 5:**
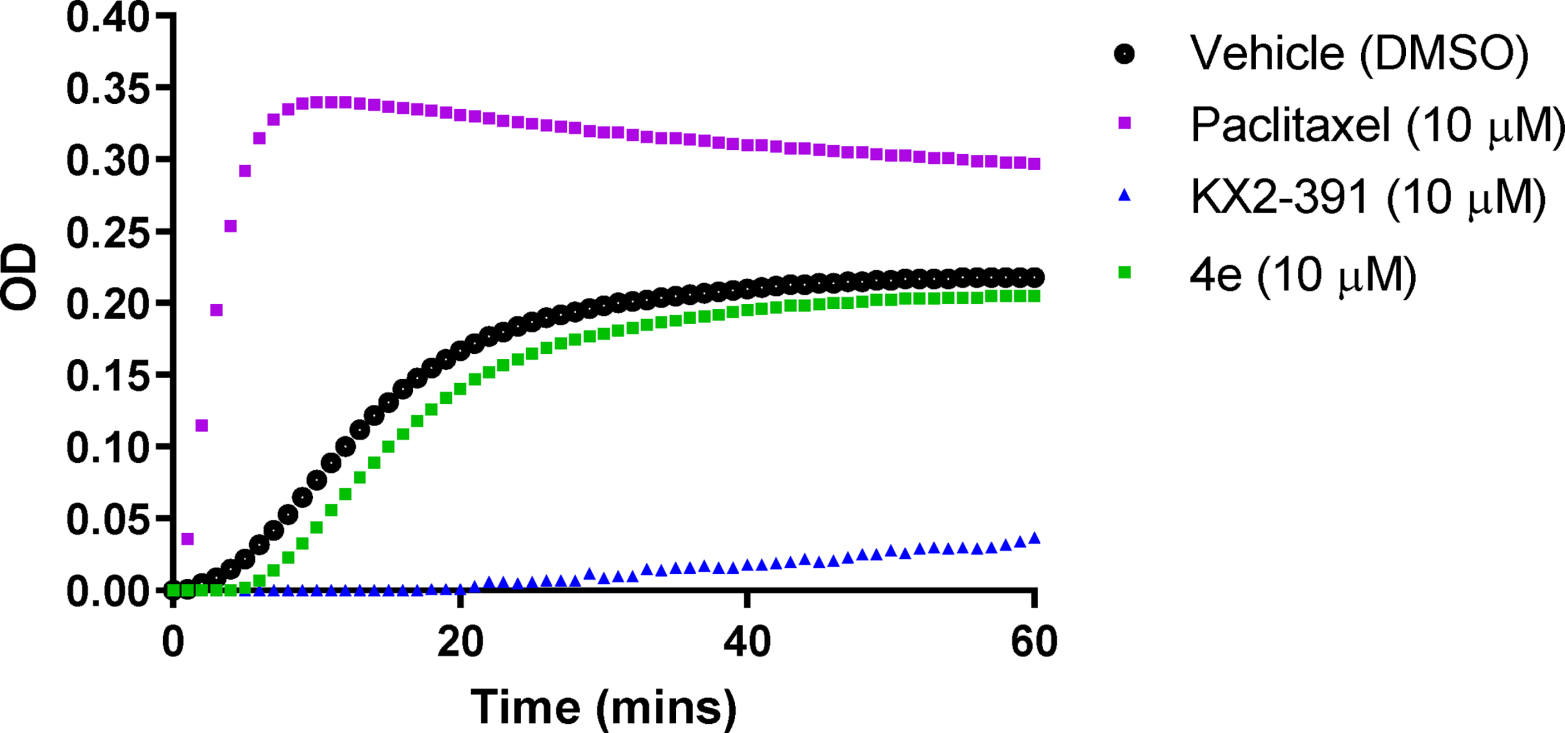
Tubulin polymerization assay for compound **4e** at 10 µM compared to paclitaxel (10 µM) and KX2-391 (10 µM) as references, while DMSO (1% v/v) was used as a vehicle control. Purified bovine tubulin and GTP were mixed in a 96-well plate. The polymerization reaction was initiated by warming the solution from 4°C to 37°C. The effect on tubulin assembly was monitored by measuring the optical density (OD) at 340 nm every 30 seconds over 60 min period. The results represent the mean for three separate experiments.

The effect of compound **4e** on tubulin polymerization was investigated using the cell-free assay which assesses the assembly of purified bovine tubulin by a turbidometric method (**Figure 5**). Tubulin polymerization results in increased turbidity, which is measured as an increase in the optical density (OD). KX2-391, a known tubulin polymerization inhibitor, was used as a positive control, while paclitaxel was used to demonstrate an effective tubulin polymer stabilizing activity (27). Compound **4e** did not affect the tubulin polymerization at a theoretical concentration of 10 µM. Since this compound showed limited solubility (< 2.0 µM in 2% DMSO/PBS), we decided against testing higher concentrations and assumed that the maximum concentration was achieved in attempting to make the compound up to the 10 mM concentration.

#### 2.3.6 Effect of 4e on ABCB1 Efflux Function

**Figure 6:**
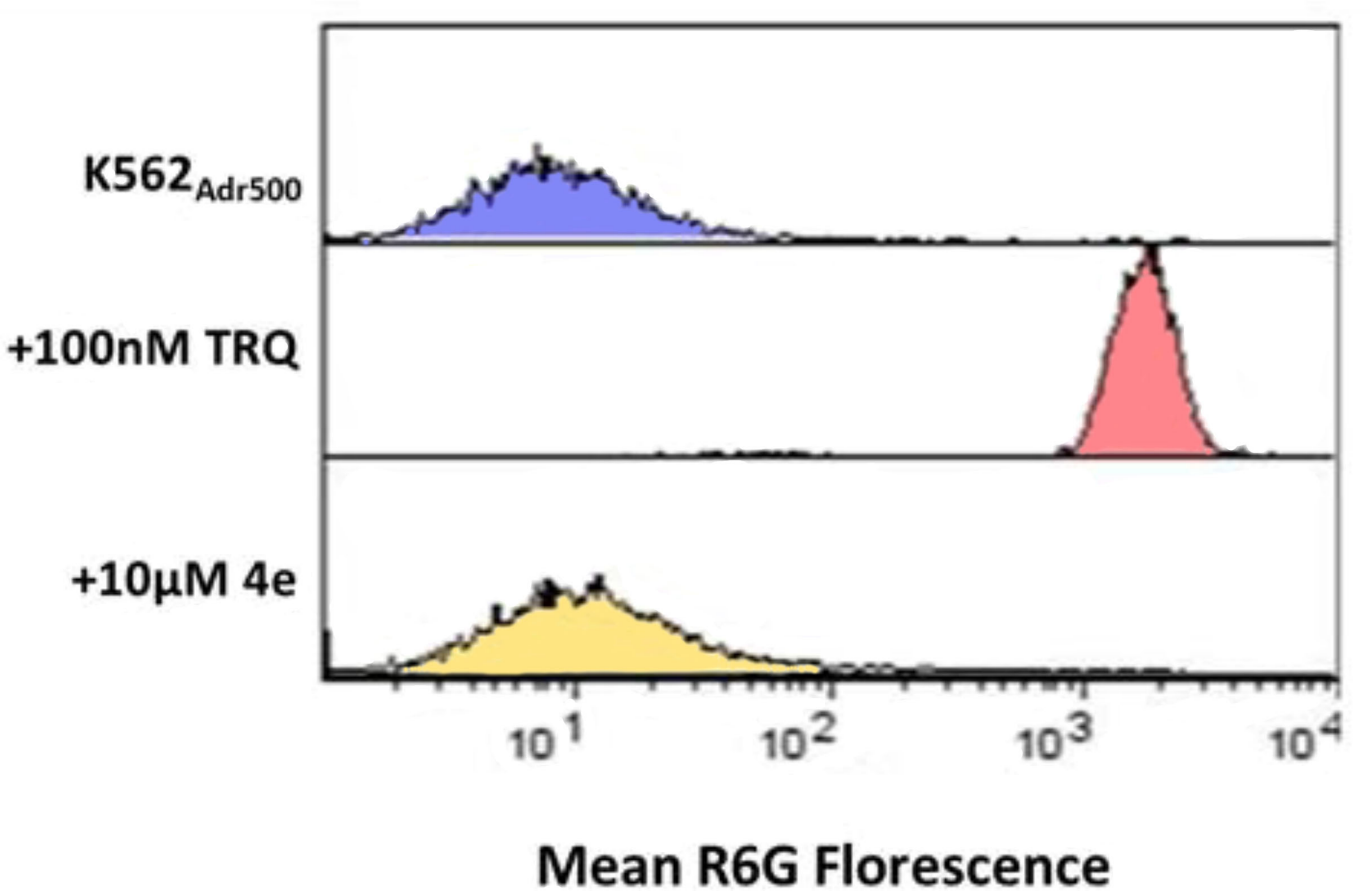
Effect of **4e** on R6G retention in K562Adr500 cells. The ABCB1 efflux activity was assessed by measuring the percentage of R6G retention in K562Adr500 cells by flow cytometry with tariquidar as the positive control.

A number of cancers acquire resistance to drug treatment by overexpressing the ATP Binding Cassette transporter ABCB1 (p-glycoprotein) (28). To understand the contribution, if any, of p-glycoprotein associated efflux on the anti-cancer activity of **4e**, we examined the interaction of **4e** with p-glycoprotein in Adriamycin resistant K562 cells (K562_Adr500_), previously shown to overexpress ABCB1 protein using rhodamine 6G dye (R6G), a florescent substrate of ABCB1. As shown in **Figure 6**, **4e** did not increase the mean R6G florescence at 10 µM concentration when compared to the untreated control. Tariquidar (TRQ), a specific ABCB1 inhibitor, showed maximum R6G retention and hence florescence at 100 nM, and was used as the positive control. The results indicate that **4e** does not interact with and modulate the activity of the ABCB1 protein and thus, is not a potential substrate of p-glycoprotein.

#### 2.3.7 Phosphotyrosine-Kinase Activity Profiling of 4e With HL60 Leukemia Cell Line

**Figure 7:**
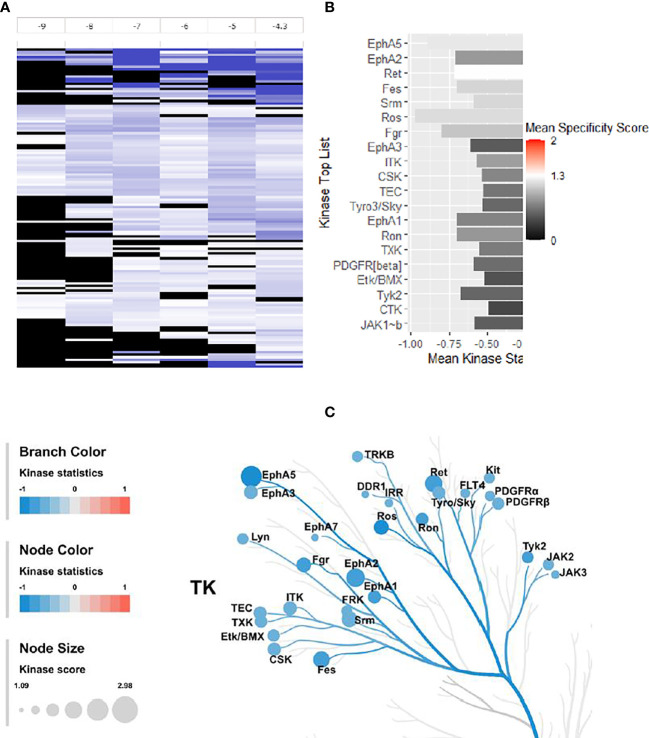
Effect of **4e** (0.1 µM) on the 144 YKs loaded on the Pamgene’s PamChip. **(A)** The LFC heatmap for 144 peptides shows the values of differentially inhibited phosphorylated peptides (treatment vs. DMSO control). The treatment effects are log-ratios (log2(treatment)-log2(control)). Black color are peptides which did not pass the threshold of inhibition. DMSO conc. 2% v/v; Compound concentrations: log -9 = 0.001 μM, log -8 = 0.01 μM, log -7 = 0.1 μM, log -6 = 1 μM, log -5 = 10 μM and log -4.3 = 50 μM. **(B)** Mean Kinase Statistic (represented by bar length) and Mean Specificity Score (represented by color scheme) for the top twenty affected YKs. **(C)** Kinome Tree: Top predicted kinases are represented on phylogenetic tree of the human protein kinase family. Size of dot indicates total kinase score, and color denotes kinases statistics (Red: higher in on-chip treated lysates, Blue: lower in on-chip treated lysate). Kinase score cut-off ≥ 1.2 was applied to select top altered kinases. Note that a file containing all kinase score above 0.0 at different concentrations can be found in **Supplementary Material** file inventory.

The changes in kinase activities induced by treatment with **4e** were monitored within the HL60 cell lines using PamGene’s (Hertogenbosch, Netherlands) PamChip^®^ peptide microarray profiler, which quantifies the activities of 144 tyrosine kinases (YK) (29, 30) (**Figure 7**). Results are provided as a log fold change (LFC) heatmap and Mean Kinase Statistic. From the LFC it is apparent that compound **4e**, at a concentration of 0.1 µM, induced sizable changes to the activity of several kinases within the cell signaling YK profile (**Figure 7A**).

An Upstream Kinase tool was used to generate a putative list of kinases responsible for phosphorylating the phospho-sites on the PamChip (**Figure 7B**) (31). It is an interpretation and is highly dependent on the contents of the underlying phosphorylation databases. A Kinase Score was used for ranking kinases based on their significance and specificity in terms of the set of peptides used for the corresponding kinase. Kinase Statistics indicates the overall change of the peptide set that represents the kinase. Kinase statistics value <0 indicates lower kinase activity in the compound spiked lysate and *vice versa* (32, 33). **Table 5** lists the 20 most changed kinases within treated HL60 cell lysates by 0.1 μM **4e**, ranked according to their Kinase Score. Results revealed that the most highly affected kinases after treatment by **4e** were Eph family kinases (EphA5, 2, and 3) and TEC family kinases (ITK, TEC, TXK and ETK/BMX).

**Table 5:** Top 20 affected kinases ranked according to Kinase Scores for **4e** at 0.1 µM treatment of HL60 leukemia cell lines.

Kinase Uniprot ID	Kinase Name	Mean Specificity Score	Mean Significance Score	Mean Final Score*	Mean Kinase Statistic
P54756	EphA5	1.1979	1.7474	2.9453	-0.8931
P29317	EphA2	0.8197	1.5807	2.4004	-0.7066
P07949	Ret	1.2940	1.0953	2.3893	-0.7112
P07332	Fes	1.1101	1.2140	2.3241	-0.6958
Q9H3Y6	Srm	1.1174	1.1518	2.2692	-0.5863
P08922	Ros	1.1413	0.9578	2.0991	-0.9704
P09769	Fgr	1.0335	1.1688	2.2023	-0.7971
P29320	EphA3	0.5017	1.4547	1.9564	-0.6074
Q08881	ITK	0.8451	1.0723	1.9173	-0.5666
P41240	CSK	0.7249	1.2107	1.9356	-0.5346
P42680	TEC	0.6383	1.3350	1.9733	-0.5256
Q06418	Tyro3/Sky	0.5745	1.2459	1.8204	-0.5274
P21709	EphA1	0.7147	1.1254	1.8401	-0.6954
Q04912	Ron	0.8144	1.0254	1.8398	-0.6967
P42681	TXK	0.6772	1.2373	1.9145	-0.5527
P09619	PDGFR[beta]	0.5995	1.2570	1.8565	-0.5885
P51813	Etk/BMX	0.4561	1.3079	1.7640	-0.5143
P29597	Tyk2	0.5624	1.1411	1.7035	-0.6710
P42679	CTK	0.3920	1.1881	1.5802	-0.4874
P23458	JAK1~b	0.5171	0.8736	1.3907	-0.5828

*Ranking was based on this score.

**Figure 8:**
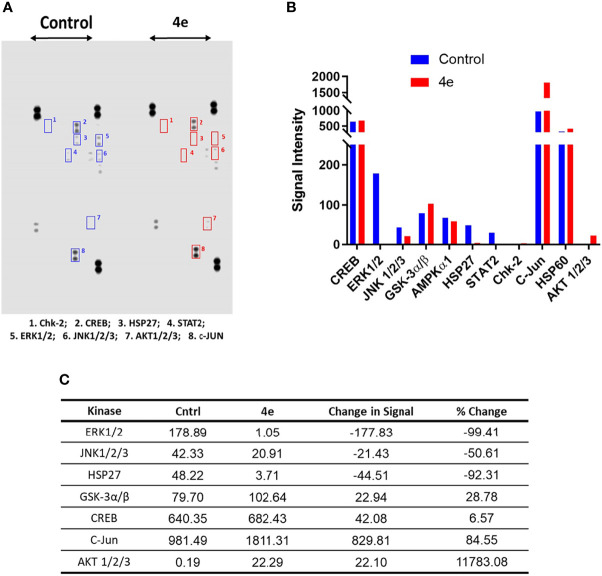
Phosphokinase YK/STK proteome profile of **4e** in HL60 cell line. **(A)** Blot scanning of detected phosphokinase proteins captured on nitrocellulose membranes pre-spotted with different antibodies for control and compound-treated HL60 cell lysates. ***(*B*)*** Bar graph quantifying the variations in YK/STK phosphokinase signals upon **4e** exposure (red bars) compared to the untreated control (blue bars). A file containing all phospho-kinase signal intensities can be found in **Supplementary Material**. **(C)** Difference in the kinase signals between treated and untreated cells.

The kinome tree representation (**Figure 7C**), which was generated by raising the Kinase Score cut-off to >1.2, confirmed that, unlike tirbanibulin and our prototype molecule KX1-136, **4e**’s effects were more observable on kinases other than the Src family such as the Eph and TEC families. However, **4e** did not show high specificity to any of the 144 YK’s (Specificity Scores >1.3, **Figure 7B**). Therefore, we screened compound **4e** using the Human Phospho-Kinase Array^®^ kit from R&D systems (Minneapolis, MN, USA). In this system, 35 phosphorylated YK and serine-threonine kinase (STK) enzymes in the untreated (control) and treated (test) HL60 cell lysates were captured on nitrocellulose membrane, pre-spotted with phosphokinase-specific capture antibodies, and then visualized by detection antibody cocktails (**Figure 8**). The difference in the spot intensity between untreated (control) and treated cells is proportional to change in the amount of the phosphorylated protein induced by **4e**.

In the Human Phospho-Kinase Array^®^ we considered the intensity of the signal in the control as an indicator for the involvement of this enzyme in HL60 proliferation. Interestingly, the Src signal was undetectable in the control (untreated) HL60 cell lysates. However, a number of signaling kinases were affected significantly in **4e** treated cell lysates. Several STK kinases were suppressed, such as the MAPK family kinases ERK1/2 and JNK. The kinase c-Jun, which already showed intense signal in the control, was profoundly upregulated by treatment of **4e**. Chk2 and AKT1/2/3 phosphorylation, which was very weak in the control cells, was enhanced in **4e** treated cell lysates, but the end signals were still weak compared to others such as c-Jun and CREB. The signal for phosphorylated ERK1/2 was modest in control cells, but was almost gone after treatment with **4e,** implying a possible role in the antiproliferative activities of the compound.

## 3 Discussion

The insertion of an amide group (a resourceful common group in drug discovery) between the two aromatic rings of KX1-136 (a confirmed non-ATP Src inhibitor) resulted in novel compounds that maintained comparable levels of anticancer activities. Compound **4n** showed, on average, the highest degree of anticancer activity, followed by **4e**. Since our phenotypic screening aimed to discover new leads that eventually can be developed as new drugs against malignant tumors, we subjected these two compounds to a battery of tests for their drug likeness. Compound **4n** showed poor stability to oxidative metabolism in mouse liver microsomes. The compound **4n** was also unstable in mouse microsomes in the absence of NADPH as well as in mouse plasma, indicating the likelihood of enzymatic hydrolysis. More important, the compound 4e demonstrated higher selectivity in leukemia cell growth inhibition over normal (non-cancerous) cell inhibition. This made it clear that 4e should be our selection for further investigations.

Both compounds **4e** and **4n** were stable in corresponding human preparations and showed no significant inhibition of the three major metabolizing human CYP450 enzymes (3A4, 2D6, and 2C9) at concentrations up to 10 mM. Both compounds demonstrated low to moderate maximum kinetic solubility in 2% DMSO/PBS but compound **4e** was somewhat more soluble than **4n**. The limited aqueous solubility seen with this scaffold to date will need to be optimized further before advancing any analog to development, but the solubility of **4e** was sufficient for our mechanistic studies. Therefore, we selected **4e** as a molecular probe to explore possible mechanisms that may differentiate our new scaffold from the parent KX chemotype.

The kinome profile of **4e** proved to be interesting. The PamChip^©^ test indicated that **4e** affected members of Eph family kinases (EphA5, 2 and 3) and TEC family kinases (ITK, TEC, TXK and ETK/BMX), not the Src kinase family. However, low Kinase Score (<1.3 according to PamGene scale, **Figure 7B**) indicated that this test was not conclusive. PamGene test uses a complex scoring process (Kinase Statistic and Kinase Score) based on networking in upstream and downstream activities for each kinase (34). Although this process may help to focus on some probable targets, results are not completely conclusive in case of non-ATP competitive inhibitors which may inhibit kinases by other means instead of phosphorylation. This result was surprising success because compound **4e** was originally inspired from the recent selective Src inhibitor KX1-136. Nevertheless, there are evidences that **4e** downregulate multiple oncogenic serine/threonine kinases and observed that it dramatically suppressed the mitogen-activated protein kinase (MAPK) family kinases (ERK1/2, and JNK), and enhanced the phosphorylation of c-Jun. Phosphorylation of c-Jun at two serine residues, Ser63 and Ser73, is known to increase c-Jun activity (35). We believe that the increased c-Jun activity is one of the major contributors to the extensive apoptosis observed during compound **4e** treatment. These results are in accordance with previous published reports that established the apoptotic capabilities of phosphorylated c-Jun in variety of cancer types (36–38).

The MAPK family kinases suppressed by **4e**, such as Extracellular signal-Regulated Kinases 1/2 (ERK1/2), are members of the Ras-Raf-Mek-Erk pathway (MAPK pathway) and are involved in cell cycle progression from G1 to S phase (39–41) as well as cell survival (42, 43). Compound **4e** caused almost complete inhibition of ERK1/2 phosphorylation on the residues Thr202/Tyr204 and Thr185/Tyr187 (only 1.6% phosphorylation compared to control). Another notable oncogenic kinase family, the active c-Jun N-terminal Kinases 1/2/3 (JNK1/2/3), promotes cell cycle progression and survival (44). JNK kinase phosphorylation levels at Thr183 and Thr85 were suppressed by treatment with **4e** by approximately 50%. Heat shock protein 27 (Hsp27) is an ATP-independent molecular chaperone whose role is to correct protein misfolding and is involved in tumorigenesis and metastasis. Hsp27 is overexpressed and phosphorylated when cells are under stress, thereby protecting the cells by activating survival mechanisms and suppressing apoptosis. In many cancer types, Hsp27 is overexpressed and interferes with apoptosis, which explains its involvement in tumorigenesis and metastasis and contribution to resistance to chemotherapy (45, 46). After **4e** treatment, phosphorylation of Hsp27 Ser78 and Ser82 was dramatically decreased by 92.3%. Glycogen synthase kinase-3α/β (GSK-3α/β) has two phosphorylation sites: the activating Tyr216, and the Akt-mediated deactivating phosphorylation at Ser9 (47, 48). Compound **4e** caused 28% increase in Ser9 phosphorylation, which contributes to suppression of the anti-apoptotic proteins’ expression (49). However, in some cancer types, including AML, inactivated GSK-3β (through increased Ser9 phosphorylation) leads to the opposite outcome where it has been associated with low overall survival of patients (50, 51). In this case, the outcome of GSK-3β/inactivation through phosphorylation at Ser9 can be considered inconclusive, and further investigation is required to decipher the true effect of GSK-3β inhibition. The protooncogene, cAMP response element-binding protein (CREB), mediates cancer progression and metastasis when it is phosphorylated at Ser133 (52). Exposure of HL60 to **4e** resulted in a minor (insignificant) increase in CREB phosphorylation (~6%). Thus, it is reasonable to hypothesize that the cytotoxic effect of compound **4e** is due to the combination of almost complete suppression of the activity of Erk1/2 and Hsp27, as well as the reduced phosphorylation of JNK1/2/3 kinases.

Our results also showed that c-Jun phosphorylation at Ser63 increased by 84% following treatment with **4e**. While phosphorylation at the C-terminus deactivates the transcription factor c-Jun, direct phosphorylation of Ser63 at the N-terminal activates the kinase (53, 54), promoting the expression of genes that inhibit apoptosis and thereby allowing cancer cell progression (54). Phosphorylation of c-Jun at this site is mainly due to the activity of JNK, which is surprising considering that JNK phosphorylation was 50% lower than that of the control after exposure to **4e**.

Additionally, the kinase profiling assay for **4e** showed high levels of phosphorylated Akt1/2/3, with >117-fold increased phosphorylation at Thr308 and Ser473 compared to control (no treatment) (55, 56). Akt (aka protein kinase B, “PKB”) is a member of the PI3K-Akt-mTOR pathway, and is implicated in cell survival, growth, proliferation, and protein synthesis through interference with phosphorylation and expression of pro-apoptotic proteins, inactivating cell cycle inhibitors, and phosphorylation of mTOR. Also, Akt1/2/3 has been reported to be over activated in acute myeloid leukemia (AML) (56–59). These findings suggest that the limited cytotoxic activity seen with **4e** in HL60 cells might have been the result of the over-activation of Akt1/2 due to treatment with the compound.

It has been established that there is crosstalk between the PI3K-Akt-mTOR and the Ras-Raf-Mek-Erk pathways which might explain the phosphorylation pattern of the key kinases in both Akt1/2/3 and Erk1/2 proteins. Some of the interactions between these pathways include a) PI3K-mediated Ras activation leading to Erk pathway activation, b) direct and indirect Erk-mediated mTOR activation, c) Akt-mediated Raf inhibition resulting in Erk/MAPK pathway inhibition, and d) inhibition of MEK by PDK1 (the upstream kinase that activates Akt1/2/3) (60). It has also been reported that MEK inhibition stops the negative feedback inhibition of the membrane RTKs, such as insulin-like growth factor 1 receptor (IGF-1R), leading to increased phosphorylation and activation of Akt1/2/3 (18, 59). It is possible that the observed shutdown of Erk1/2 activity is mediated by direct inhibition of Erk1/2 and/or its upstream kinases by **4e**, or through the multiple inhibitory crosstalk points with PI3K-Akt-mTOR pathway components, such as indirect inhibition of MEK by PDK1 (the Akt upstream kinase), or indirect suppression of Raf by the over-active Akt1/2/3 (60).

The two multiple-kinase tests (PamChip and Phosphokinase Profiler) provided strong evidence of very minor involvement of Src in the cellular activities of **4e**. However, the question must be raised about the effect of the **4e** on other SRC family kinase (SFK). To address this point, we must highlight that both R&D phosphokinase profiler and PamChip feature Yes, Fgr, Lyn, Lck and Src while Fyn, Hck, Blk and Frk activities are tested in the PamChip only. The signals of SFKs in the R&D Phosphokinase Profiler were too weak to detect in both untreated (control) and **4e**-treated HL60 cells. In PamChip assay, most of SFK’s were not ranked in the top 20 affected kinases (**Table 5**). In addition, their Specificity Score were very low (**Table 6**) indicating their marginal importance to the cell killing activities of **4e**. Fgr kinase which ranked 7^th^ in the list (**Table 5**) was the only SFK member that seems to be affected by the compound, albeit with low Specificity Score (1.03). Furthermore, Fgr signal was not detected in R&D Phosphokinase profiler in the control, indicating that Fgr is evidently a less likely target for **4e**.

**Table 6:** Effect of **4e** (0.1 µM) on Src family kinases activities within HL60 cells.

Kinase Uniprot ID	Kinase Name	PamGene Ranking	Mean Specificity Score	Mean Significance Score	Mean Final Score	Mean Kinase Statistic
P09769	Fgr	7	1.0335	1.1688	2.2023	-0.7971
P07948	Lyn	21	0.6815	1.2715	1.9530	-0.5775
P42685	Frk	26	0.5102	1.1484	1.6587	-0.5116
P06241	Fyn	27	0.3458	1.1307	1.4765	-0.4963
P06239	Lck	37	0.4082	0.8208	1.2290	-0.5054
P07947	Yes	43	0.1390	1.0126	1.1516	-0.4139
P08631	Hck	46	0.2624	0.8248	1.0872	-0.4497
P12931	Src	62	0.0236	0.7353	0.7589	-0.3527
P51451	Blk	63	0.0736	0.6625	0.7362	-0.3552

While these data are preliminary and additional studies are required to draw definitive conclusions, our findings have provided some understanding of the behavior of **4e** and how it affects the intracellular components of cancer cells. These results serve as a guide for developing analogues of compound **4e** and indicate that anticancer activity can be achieved without the activation of compensatory kinases that are known to facilitate cancer cell survival.

Compound **4e** showed inhibitory activities against a variety of tyrosine and serine/threonine kinases and anti-proliferative activity in a number of cancer cell lines that recommend further study of the scaffold for the treatment of cancer. In the clinic, cancers have already developed resistance to approved Src kinase inhibitors, primarily through their ability to activate a panel of survival kinases like ERK1/2, PI3K/Akt and mTOR (61–64), requiring the need for combinational therapies. Since compound **4e** shows robust inhibition of ERK1/2, in addition to its anticancer mechanisms, we believe that compounds like **4e** will have advantages compared to preexisting Src kinase inhibitors by being able to inhibit the activation of the compensatory kinases responsible for resistance. However, additional studies need to be done to confirm these hypotheses. Those studies will be the subject of future publications.

In conclusion, this research proved that *N*-benzyl-2-aryacetamide is a promising pharmacophore for developing new anticancer agents that is not specified to target Src or SFK only. Altering the substitution around this scaffold by amide group, for instance, weakened that effect on both Src and tubulin and depended on other targets.

## 4 Materials and Methods

### 4.1 Chemical Synthesis

All melting points were uncorrected and measured using the capillary melting point instrument BI 9100 (Barnstead Electrothermal, UK). ^1^H NMR spectra were determined on an AVANCE-III 600 MHz and AVANCE-III HD 850 MHz spectrometers (Bruker, Germany), and chemical shifts were expressed as ppm against TMS as an internal reference (King Fahd Center for Medical Research and Faculty of Science, King Abdulaziz University, Jeddah, Saudi Arabia). LC/MS analyses were performed on an Agilent 6320 Ion Trap HPLC–ESI-MS/DAD (Santa Clara, CA, USA) with the following settings: The analytes were separated using an Macherey-Nagel Nucleodur-C18 column (150 mm length × 4.6 mm i.d., 5 µm) (Macherey-Nagel GMBH & Co. KG, Duren, Germany). Mobile phase; isocratic elution using a mixture of acetonitrile and 0.01 formic acid in water (80: 20, v/v). The flow rate was 0.4 mL/min; total run time = 20 min. Purities are reported according to percentage of Peak Areas at wavelength 280 nm. High-resolution mass spectrometry (HRMS) was performed in the Faculty of Science, King Abdulaziz University on Impact II™ Q-TOF spectrometer (Bruker, Germany). LCMS and HRMS analyses of some compounds were performed by LTQ-XL Linear Ion Trap Mass Spectrometer coupled with Accela autosampler and Accela pump, Thermo Fisher Scientific Inc, San Jose, CA, USA. The ion source; electrospray ionization compartment. The system was controlled with Xcalibur^®^ Thermo Fisher Scientific Inc, v.2.07 SP1. Spay voltage, 5.0 kv, sheath gas flow rate, 45 mL/min, auxiliary gas, 10 mL/min, sweep gas, 5 mL/min, capillary voltage, 60v, capillary temperature, 320°C, scan range, +100 - 700 *m/z*. The collision energy was 35 v. Column, Eclipse Plus C18, 3.5 µm, 4.6 ×100 mm (Agilent, Palo Alto, USA), column oven, 25°C. Tray temperature 20°C. The mobile system was composed of (A) acetonitrile and (B) water containing 5 mM ammonium acetate, 1 mL glacial acetic acid 100%. The flow rate was 400 μL/min. The injection volume was 5 μL. The pump was programmed to deliver 65%A: 35%B. Data was confirmed by the aid of NIST 2017, using MS interpreter software version BETA 3.1A build 05/01/2017. HRMS for most compounds were performed by LTQ-XL Linear Ion Trap Mass Spectrometer and allying the same method mentioned for the LCMS analysis above. Column chromatography was performed on a silica gel 60 (particle size 0.06 mm - 0.20 mm).

#### 4.1.1 General Method for Synthesis of 2-(4-aminophenyl)-(*N*-substituted) Acetamide (3a-3c)

A mixture of 2-(4-nitrophenyl)acetic acid (10 mmol, 1.81 g) and 50 mL dichloromethane (DCM) was placed in a dry 3-neck round bottom flask and flushed by nitrogen which was then stirred in an ice bath. Oxalyl chloride (11.6 mmol, 1 mL) in 5 mL DCM was placed in an addition funnel and was then fast-dropped to the original mixture. After completing the addition of oxalyl chloride, 1 drop of *N,N-*dimethylformamide (DMF) was added. After 15 min, the ice bath was removed and the mixture was stirred at room temperature and the stirring continued for 4 h till all the acid has completely dissolved. The solvent was removed using a rotary evaporator and the residue 2-(4-nitrophenyl)acetyl chloride was dissolved in 40 mL DCM and was stirred for 10 min in an ice bath. Using an addition funnel, a mixture of the appropriate amine (10 mmol) and diisopropylethylamine (DIPEA) (10 mmol, 2.1 mL) in 40 mL DCM was added drop wise to the acid chloride. After the ice melted, the stirring continued overnight at room temperature. The solid particles were removed by filtration and washed with a small amount of DCM forming a yellowish white crystalline solid. The completion of the reaction was checked by TLC for both the solid and filtrate using ethyl acetate hexane mixture in the ratio 1:1 against the starting materials. The filtrate was neutralized by HCl (1 N), and the organic layer was collected and dried over sodium sulfate. The solvent was rotavaped and the solid was collected and washed with ether to give the 2-(4-nitrophenyl)-(*N*-substituted) acetamides (**2a-2e**). In a 150 mL round-bottom flask rapped with Aluminum foil, a mixture of each (**2a-2e**) (6.58 mmol) and SnCl_2_ dihydrate (26.34 mmol, 5.95 g) in ethyl acetate (40 mL) and water (0.5 mL) was refluxed for 4 h. The mixture was cooled and diluted by ethyl acetate (40 mL) and then treated with a cold solution of 40% NaOH (80 mL), forming an emulsion, then fresh water was added to break it successfully. The organic layer was separated then the aqueous layer was washed with 10 ml of ethyl acetate; the combined organic layers were washed with 15 ml of brine and dried with magnesium sulfate. The ethyl acetate was distilled off using the rotary evaporator and the solid products were collected as the 2-(4-aminophenyl)-(*N*-substituted) acetamides (**3a-3e**) intermediates.

##### 4.1.1.1 2-(4-aminophenyl)-N-benzylacetamide (3a)

This amine was synthesized using benzyl amine according to the procedure above in the general method, and its characterization data were found similar to literature (65). The reaction yielded 1.55 g (98.7%) of solid. Melting point 140-141°C. ^1^H NMR (DMSO-*d*_6_, 850 MHz) δ 8.35 (br t, 1H, *J*=5.7 Hz), 7.30 (t, 2H, *J*=7.4 Hz), 7.2-7.2 (m, 3H), 6.9-6.9 (m, 2H, *J*=8.3 Hz), 6.5-6.5 (m, 2H, *J*=8.3 Hz), 4.90 (s, 2H), 4.25 (d, 2H, *J*=6.0 Hz), 3.26 (s, 2H). LC-MS (ESI), RT = 1.7 min; *m/z* 241.8 [M+H]^+^. The compound was used to the next step without further characterization.

##### 4.1.1.2 2-(4-aminophenyl)-N-phenethylacetamide (3b)

This amine was synthesized using 2-phenylethan-1-amine according to the general method. The reaction yield was 71.3% of solid. Melting point 123-126.4°C. ^1^H NMR (600 MHz, CDCl_3_) δ 7.17 - 7.34 (m, 3H), 7.05 (d, *J* = 7.53 Hz, 2H), 6.94 (d, *J* = 8.28 Hz, 2H), 6.63 (d, *J* = 7.91 Hz, 2H), 5.43 (br. s., 1H), 3.70 (br. s., 1H), 3.38 - 3.56 (m, 4H), 2.68 - 2.81 (m, 2H). The compound was used to the next step without further characterization.

##### 4.1.1.3 2-(4-aminophenyl)-N-(furan-2-ylmethyl)acetamide (3c)

This amine was synthesized using furan-2-ylmethanamine according to the general method. The reaction yield was 42.6%. Melting point 162.5-165.7°C. ^1^H NMR (600 MHz, CDCl_3_) δ 7.02 (d, *J* = 8.28 Hz, 2H), 6.65 (d, *J* = 8.28 Hz, 2H), 6.28 (br. s., 1H), 6.13 (br. s., 1H), 5.78 (br. s., 1H), 4.38 (d, *J* = 5.65 Hz, 2H), 3.70 (br. s., 2H), 3.43 - 3.60 (m, 2H). The compound was used to the next step without further characterization.

#### 4.1.2 Procedures for the Synthesis of Compounds 4a—4s:

##### 4.1.2.1 2-(4-acetamidophenyl)-N-benzylacetamide (4a)

In an ice bath, the acetyl chloride was dissolved in 75 mL DCM and stirred for 10 min. Using addition funnel, 2-(4-aminophenyl)-*N*-benzylacetamide (**3a**) (2 mmol, 0.48 g) and Diisopropylethylamine (DIPEA) (10 mmol, 2.1 mL) in 25 mL DCM were added drop wise to the acetyl chloride. After the ice melted, the stirring continued over night at room temperature. The completion of the reaction was checked by TLC for both the solid and filtrate using the ethyl acetate hexane mixture in the ratio 1:1 against starting materials. The mixture was neutralized by dil. HCl, and the product was extracted with ethyl acetate. The organic layer was washed, collected, and dried using sodium sulfate. The solvent was rotavaped and the solid was collected and washed with ether. The product was purified by crystallization from hot methanol. Melting point of the final product is 175°C. ^1^H NMR (600 MHz, DMSO-*d*_6_) δ ppm 9.92 (br. s., 1H), 8.51 (br. s., 1H), 7.50 (d, *J* = 7.91 Hz, 2H), 7.28 - 7.36 (m, 2H), 7.16 - 7.27 (m, 4H), 4.23 - 4.31 (m, 2H), 3.42 (br. s., 2H), 2.04 (br. s., 3H). ^13^C NMR (20 MHz, DMSO-*d*_6_) δ 170.3, 168.5, 166.0, 164.1, 150.6, 139.5, 137.6, 133.5, 133.1, 131.5, 131.0, 129.1, 128.3, 127.9, 127.2, 126.8, 120.2, 55.5, 20.4, 19.5. LC-MS (ESI), RT = 1.7 min; *m/z* 283.5 [M+H]^+^.

##### 4.1.2.2 N-(4-(2-(benzylamino)-2-oxoethyl)phenyl)propionamide (4b)

The compound was synthesized by reacting propionyl chloride with **3a** according to the procedure described for the synthesis of **4a**. The compound was white solid (23%). Melting point 210°C. ^1^H NMR (850 MHz, DMSO-*d*_6_) δ 9.83 (s, 1H), 8.51 (br. s., 1H), 7.50 (d, *J* = 7.78 Hz, 1H), 7.27 - 7.33 (m, 2H), 7.20 - 7.26 (m, 3H), 7.18 (d, *J* = 7.78 Hz, 2H), 4.26 (d, *J* = 5.71 Hz, 2H), 3.41 (s., 2H), 2.30 (q, *J* = 7.27 Hz, 2H), 1.07 (t, *J* = 7.53 Hz, 2H). ^13^C NMR (214 MHz, DMSO-*d*_6_) δ 172.4, 170.9, 139.9, 138.2, 131.3, 129.7, 128.7, 127.6, 127.2, 119.4, 42.6, 42.2, 29.9, 10.2. LC-MS (ESI), RT = 1.9 min; *m/z* 297.4 [M+H]^+^.

##### 4.1.2.3 N-(4-(2-(benzylamino)-2-oxoethyl)phenyl)benzamide (4c)

This compound **4c** was prepared by reacting benzoyl chloride with **3a** according to the procedure described for the synthesis of **4a**. The compound was white solid (95%). Melting point 212°C. ^1^H NMR (850 MHz, DMSO-*d*_6_) δ 10.23 (s, 1H), 8.55 (t, *J* = 5.71 Hz, 1H), 7.95 (d, *J* = 6.75 Hz, 2H), 7.67 - 7.71 (m, *J* = 8.30 Hz, 2H), 7.59 (t, *J* = 7.53 Hz, 1H), 7.53 (t, *J* = 7.53 Hz, 2H), 7.29 - 7.34 (m, 2H), 7.25 - 7.27 (m, *J* = 8.30 Hz, 2H), 7.22 - 7.25 (m, 3H), 4.27 (d, *J* = 5.71 Hz, 2H). ^13^C NMR (214 MHz, DMSO-*d*_6_) δ 170.8, 166.0, 139.9, 138.0, 135.4, 132.2, 132.0, 129.6, 128.9, 128.8, 128.1, 127.7, 127.3, 120.8, 42.7, 42.3. LC-MS (ESI), RT = 2.0 min; *m/z* 345.2 [M+H]^+^.

##### 4.1.2.4 N-(4-(2-(benzylamino)-2-oxoethyl)phenyl)-3-fluorobenzamide (4d)

This compound was prepared by reacting 3-fluorobenzoyl chloride with **3a** according to the procedure described for the synthesis of **4a.** The compound’s yield was 89%. Melting point 214°C. ^1^H NMR (850 MHz, DMSO-*d*_6_) δ 10.30 (s, 1H), 8.55 (t, *J* = 5.97 Hz, 1H), 7.81 (d, *J* = 7.79 Hz, 1H), 7.76 (td, *J* = 2.21, 9.60 Hz, 1H), 7.66 - 7.70 (m, *J* = 8.30 Hz, 2H), 7.59 (dt, *J* = 5.97, 7.91 Hz, 1H), 7.45 (dt, *J* = 2.34, 8.69 Hz, 1H), 7.29 - 7.33 (m, 2H), 7.25 - 7.29 (m, *J* = 8.30 Hz, 2H), 7.21 - 7.25 (m, 3H), 4.27 (d, *J* = 5.71 Hz, 2H), 3.51 (br. s., 1H). ^13^C NMR (214 MHz, DMSO-*d*_6_) δ 170.8, 164.5, 164.5, 163.0, 161.8, 139.9, 137.7, 137.6, 132.4, 131.1, 131.1, 129.7, 128.8, 127.7, 127.3, 124.3, 124.3, 120.9, 119.0, 118.9, 114.9, 114.8, 42.7, 42.3. LC-MS (ESI), RT = 2.1 min; *m/z* 363.2 [M+H]^+^.

##### 4.1.2.5 N-(4-(2-(benzylamino)-2-oxoethyl)phenyl)-3-chlorobenzamide (4e)

This compound was prepared by reacting 3-chlorobenzoyl chloride with **3a** according to the procedure described for the synthesis of **4a**. The compound was white solid (97.6%). Melting point 207°C. ^1^H NMR (850 MHz, DMSO-*d*_6_) δ 10.34 (s, 1H), 8.56 (d, *J* = 5.71 Hz, 1H), 8.02 (t, *J* = 1.82 Hz, 1H), 7.93 (d, *J* = 8.30 Hz, 1H), 7.68 - 7.71 (m, *J* = 8.30 Hz, 2H), 7.66 - 7.68 (m, 1H), 7.57 (t, *J* = 8.04 Hz, 1H), 7.30 - 7.33 (m, 2H), 7.26 - 7.28 (m, *J* = 8.30 Hz, 2H), 7.24 (d, *J* = 7.27 Hz, 2H), 4.28 (d, *J* = 6.23 Hz, 2H), 3.47 (s, 2H); ^13^C NMR (214 MHz, DMSO-*d*_6_) δ 170.7, 164.4, 139.9, 137.7, 137.4, 133.7, 132.5, 131.8, 130.9, 129.7, 128.8, 127.9, 127.7, 127.6, 127.2, 126.9, 120.9, 42.7, 42.3. LC-MS (ESI), RT = 1.1 min; *m/z* 379.1 [M+H]^+^. HRMS (ESI), RT= 4.21 min, *m/z* 379.36285 [M + H]^+^, formula C_22_H_19_ClN_2_O_2_.

##### 4.1.2.6 N-(4-(2-(benzylamino)-2-oxoethyl)phenyl)-4-chlorobenzamide (4f)

This compound was prepared by reacting 4-chlorobenzoyl chloride with **3a** according to the procedure described for the synthesis of **4a**. The compound was white solid (73.8%). Melting point 190°C. ^1^H NMR (850 MHz, DMSO-*d*_6_) δ 10.29 (s, 1H), 8.53 (br t, 1H, *J*=5.7 Hz), 7.99 (d, 2H, *J*=8.6 Hz), 7.95 (d, 1H, *J*=8.3 Hz), 7.68 (d, 2H, *J*=8.6 Hz), 7.62 (d, 2H, *J*=8.6 Hz), 7.58 (d, 1H, *J*=8.3 Hz), 7.32 (t, 2H, *J*=1.0 Hz), 7.26 (d, 2H, *J*=8.3 Hz), 7.2-7.3 (m, 3H), 4.28 (d, 2H, *J*=6.0 Hz), 3.46 (s, 3H). ^13^C NMR (214 MHz, DMSO-*d*_6_) δ 170.7, 164.8, 139.9, 137.8, 136.8, 134.1, 132.3, 131.6, 130.1, 129.7, 129.2, 128.9, 128.8, 127.7, 127.2, 120.8, 42.7, 42.3. LC-MS (ESI), RT = 2.3 min; *m/z* 379.1 [M+H]^+^.

##### 4.1.2.7 N-benzyl-2-(4-((4-methylphenyl)sulfonamido)phenyl)acetamide (4g)

This compound was prepared by reacting 4-methylbenzenesulfonyl chloride with **3a** according to the procedure described for the synthesis of **4a**. The compound was white solid (21.9%). Melting point 140°C. ^1^H NMR (850 MHz, DMSO-*d*_6_) δ 8.49 (t, *J* = 5.97 Hz, 1H), 7.63 - 7.65 (m, *J* = 8.30 Hz, 2H), 7.32 - 7.34 (m, *J* = 8.30 Hz, 2H), 7.27 (t, *J* = 7.27 Hz, 2H), 7.22 (t, *J* = 7.27 Hz, 1H), 7.17 (d, *J* = 7.27 Hz, 2H), 7.09 - 7.12 (m, *J* = 8.30 Hz, 2H), 6.98 - 7.01 (m, 2H), 4.22 (d, *J* = 6.23 Hz, 2H), 3.35 (s, 2H), 2.33 (s, 3H). ^13^C NMR (214 MHz, DMSO-*d*_6_) δ 170.6, 143.6, 139.8, 137.3, 136.8, 132.3, 130.1, 130.1, 128.7, 127.6, 127.2, 127.2, 120.4, 42.6, 42.0, 21.4. LC-MS (ESI), RT = 2.0 min; *m/z* 395.1 [M+H]^+^.

##### 4.1.2.8 N-(4-(2-(benzylamino)-2-oxoethyl)phenyl)-3-methoxybenzamide (4h)

3-Methoxybenzoic acid (3.482 g, 2.29 mmol, 1.1 eq.) and 3*H*-[1,2,3]triazolo[4,5-b]pyridin-3-ol (1.631g, 2.29 mmol, 1.1 eq.) were added to a solution of **3a** (0.5 g, 2.08 mmol, 1 eq.) in dry DMA (3 mL). The resulting mixture was cooled to 0°C, then EDCE (3.877 g, 2.5 mmol, 1.2 eq.) was added dropwise. The reaction mixture was stirred at RT overnight. Then 20 mL H_2_O was added to reaction mixture and extracted with EtOAc (2 × 15 mL). All the organic layers were combined, washed with water (3 × 10 mL), dried over Na_2_SO_4_, and concentrated under vacuum. The resulting material was triturated in *i*-PrOH/hexane, the solid precipitate was filtrated, washed *i*-PrOH then hexane, and dried in vacuum to give 0.39 g of **4h** (Yield 78%). Melting point 161°C. ^1^H NMR (400 MHz, DMSO-*d*_6_) δ 10.19 (s, 1H), 8.52 (t, *J* = 6.0 Hz, 1H), 7.70 (d, *J* = 8.1 Hz, 2H), 7.55 (d, *J* = 7.7 Hz, 1H), 7.50 (d, *J* = 2.6 Hz, 1H), 7.44 (t, *J* = 7.9 Hz, 1H), 7.31 (t, *J* = 7.4 Hz, 2H), 7.24 (q, *J* = 7.7, 6.9 Hz, 5H), 7.15 (dd, *J* = 8.2, 2.6 Hz, 1H), 4.28 (d, *J* = 5.9 Hz, 2H), 3.84 (s, 3H), 3.46 (s, 2H). ^13^C NMR (126 MHz, DMSO-*d*_6_) δ 170.69, 165.56, 159.66, 139.96, 137.94, 136.83, 132.20, 130.00, 129.60, 128.74, 127.69, 127.23, 120.87, 120.31, 117.74, 113.36, 55.82, 42.71, 42.32. LC-MS (ESI), RT = 1.296 min; *m/z* 375.20 [M+H]^+^. HRMS (ESI), RT= 6.017 min, *m/z* 375.17065 [M + H]^+^, formula C_23_H_22_N_2_O_3_.

##### 4.1.2.9 N-(4-(2-(benzylamino)-2-oxoethyl)phenyl)-3-(2-morpholinoethoxy)benzamide (4i)

K_2_CO_3_ (1.3g, 9.42mmol) was added to a solution of methyl 3-hydroxybenzoate (0.573 **g**, 3,77 mmol) in DMF (7 mL). The resulting mixture was stirred at RT for 15 min, then 4-(2-bromoethyl)morpholine (1.14 **g**, 4.15 mmol) in form of hydrobromide salt was added to the solution and stirred at 50°C for 3 h. The reaction mixture was cooled to RT, poured into cold water (50 mL) and extracted with EtOAc (3 × 30 mL). All the organic layers were combined, washed with water (2 × 20 mL), dried over Na_2_SO_4_, and concentrated under vacuum. The resulting methyl 3-(2-morpholinoethoxy)benzoate was dissolved in THF (10 mL) and then was added aqueous LiOH solution (10%, 2 mL). The resulting mixture was stirred at RT for 16 h. The reaction mixture was evaporated in vacuum to dryness, and the residue was triturated in ether, dried in vacuum and used in next stage as Li salt (0.785 g, 90%).

Then, 3-(2-morpholinoethoxy)benzoic acid Li salt was reacted with **3a** following the method used for **4h** to synthesize the target compound **4i**. The reaction produced 0.282 g (56.4%). Melting point 152°C. ^1^H NMR (400 MHz, DMSO-*d*_6_) δ 10.17 (s, 1H), 8.52 (t, *J* = 6.1 Hz, 1H), 7.70 (d, *J* = 8.1 Hz, 2H), 7.53 (d, *J* = 9.1 Hz, 2H), 7.43 (t, *J* = 7.9 Hz, 1H), 7.39 – 7.08 (m, 8H), 4.28 (d, *J* = 5.9 Hz, 2H), 4.16 (t *J* = 5.9 Hz, 2H), 3.59 (s, 4H), 3.46 (s, 2H), 2.73 (t, *J* = 5.7 Hz, 2H), 2.50 (s, 5H).^13^C NMR (126 MHz, DMSO-*d*_6_) δ 170.68, 165.48, 158.86, 139.96, 137.93, 136.75, 132.19, 130.02, 129.59, 128.74, 127.69, 127.23, 120.85, 120.43, 118.13, 114.02, 66.68, 66.04, 57.50, 54.13, 42.70, 42.32. LC-MS (ESI), RT = 1.060 min; *m/z* 474.00 [M+H]^+^. HRMS (ESI), RT= 4.733 min, *m/z* 474.23898 [M + H]^+^, formula C_28_H_31_N_3_O_4_.

##### 4.1.2.10 N-(4-(2-(benzylamino)-2-oxoethyl)phenyl)-3-chloro-4-methoxybenzamide (4j)

The compound was synthesized by reacting 3-chloro-4-methoxybenzoic acid with **3a** according to the procedure for the synthesis of **4h**. The reaction produced 0.375 g (75%). Melting point 186°C. ^1^H NMR (400 MHz, DMSO-*d*_6_) δ 10.15 (s, 1H), 8.52 (t, *J* = 6.1 Hz, 1H), 8.09 (d, *J* = 2.2 Hz, 1H), 7.98 (dd, *J* = 8.7, 2.2 Hz, 1H), 7.68 (d, *J* = 8.1 Hz, 2H), 7.39 – 6.94 (m, 8H), 4.28 (d, *J* = 5.9 Hz, 2H), 3.94 (s, 3H), 3.46 (s, 2H). ^13^C NMR (126 MHz, DMSO-*d*_6_) δ 170.68, 163.96, 157.47, 139.96, 137.91, 132.16, 129.62, 128.91, 128.74, 128.18, 127.69, 127.22, 121.34, 120.83, 112.81, 56.95, 42.70, 42.31. LC-MS (ESI), RT = 1.354 min; *m/z* 409.20 [M+H]^+^. HRMS (ESI), RT= 6.265 min, *m/z* 409.13175 [M+H]^+^, formula C_23_H_21_ClN_2_O_3_.

##### 4.1.2.11 N-(4-(2-(benzylamino)-2-oxoethyl)phenyl)-3,4-dimethoxybenzamide (4k)

The compound was synthesized by reacting 3,4-dimethoxybenzoic acid with **3a** following the method used for **4h**. The reaction yielded 0.325 g (65%) of compound **4k**. Melting point 215°C. ^1^H NMR (400 MHz, DMSO-*d*_6_) δ 10.04 (s, 1H), 8.52 (t, *J* = 6.0 Hz, 1H), 7.68 (d, *J* = 8.1 Hz, 2H), 7.63 (d, *J* = 8.5 Hz, 1H), 7.55 (s, 1H), 7.31 (t, *J* = 7.3 Hz, 2H), 7.24 (d, *J* = 6.8 Hz, 5H), 7.08 (d, *J* = 8.4 Hz, 1H), 4.28 (d, *J* = 5.9 Hz, 2H), 3.85 (s, 3H), 3.84 (s, 3H), 3.46 (s, 2H).^13^C NMR (126 MHz, DMSO-*d*_6_) δ 170.71, 165.23, 152.07, 148.78, 139.97, 138.11, 131.95, 129.56, 128.74, 127.69, 127.47, 127.23, 121.46, 120.90, 111.53, 111.37, 56.13, 42.70, 42.32. LC-MS (ESI), RT = 1.242 min; *m/z* 404.20 [M+H]^+^. HRMS (ESI), RT= 5.776 min, *m/z* 404.17465 [M+H]^+^, formula C_24_H_25_N_2_O_4_.

##### 4.1.2.12 N-(4-(2-(benzylamino)-2-oxoethyl)phenyl)-3-chloro-4-(2-morpholinoethoxy) benzamide (4l)

The 3-chloro-4-(2-morpholinoethoxy) benzoic acid Li salt starting material was synthesized following the synthetic method used for the synthesis of 3-(2-morpholinoethoxy)benzoic acid Li salt mentioned within the synthesis of **4i**. The final product **4l** was synthesized by reacting 3-chloro-4-(2-morpholinoethoxy) benzoic acid Li salt with **3a** according to the procedure used for the synthesis of **4h**. The reaction produced 0.323 g (64.6%). Melting point 147°C. ^1^H NMR (400 MHz, DMSO-*d*_6_) δ 10.14 (s, 1H), 8.50 (d, *J* = 6.4 Hz, 1H), 8.08 (s, 1H), 7.96 (d, *J* = 8.7 Hz, 1H), 7.68 (d, *J* = 8.1 Hz, 2H), 7.37 – 7.28 (m, 3H), 7.24 (d, *J* = 6.9 Hz, 5H), 4.28 (d, *J* = 5.8 Hz, 4H), 3.58 (t, *J* = 4.6 Hz, 4H), 3.46 (s, 2H), 2.76 (d, *J* = 6.0 Hz, 2H), 2.51 (s, 4H). ^13^C NMR (126 MHz, DMSO-*d*_6_) δ 170.68, 163.94, 156.78, 139.96, 137.91, 132.16, 129.64, 128.77, 128.19, 127.69, 127.22, 121.60, 120.82, 113.73, 67.79, 66.71, 57.13, 54.17, 42.70, 42.31, 40.93. LC-MS (ESI), RT = 0.964 min; *m/z* 508.2 [M+H]^+^. HRMS (ESI), RT= 4.918 min, *m/z* 508.19974 [M+H]^+^, formula C_28_H_30_ClN_3_O_4_.

##### 4.1.2.13 N-(4-(2-(benzylamino)-2-oxoethyl)phenyl)-2-chloroisonicotinamide (4m)

The compound was synthesized by reacting 2-chloropyridine-4-carboxylic acid (2-chloroisonicotinic acid) with **3a** according to the procedure for the synthesis of **4h**. The reaction produced 0.380 g (76%). Melting point 214°C. ^1^H NMR (400 MHz, DMSO-*d*_6_) δ 10.54 (s, 1H), 8.62 (d, *J* = 5.1 Hz, 1H), 8.53 (d, *J* = 6.1 Hz, 1H), 8.00 (s, 1H), 7.87 (d, *J* = 5.1 Hz, 1H), 7.69 (d, *J* = 8.1 Hz, 2H), 7.40 – 7.26 (m, 4H), 7.24 (d, *J* = 7.2 Hz, 3H), 4.28 (d, *J* = 5.9 Hz, 2H), 3.48 (s, 2H). ^13^C NMR (126 MHz, CDCl_3_) δ 170.59, 162.77, 151.31, 151.16, 145.94, 139.93, 132.98, 129.78, 128.74, 127.70, 127.23, 122.73, 121.74, 120.91, 42.71, 42.30; LC-MS (ESI), RT = 1.260 min; *m/z* 380.2 [M+H]^+^. HRMS (ESI), RT= 5.853 min, *m/z* 380.11660 [M + H]^+^, formula C_21_H_18_ClN_3_O_2_.

##### 4.1.2.14 N-(4-(2-(benzylamino)-2-oxoethyl)phenyl)-6-chloropicolinamide (4n)

The compound was synthesized by reacting 6-chloropyridine-2-carboxylic acid (6-chloropicolinic acid) with **3a** according to the procedure for the synthesis of **4h**. The reaction produced 0.293 g (58.6%). Melting point 192°C. ^1^H NMR (400 MHz, DMSO-*d*_6_) δ 10.38 (s, 1H), 8.53 (t, *J* = 6.0 Hz, 1H), 8.12 (s, 2H), 7.93 – 7.70 (m, 3H), 7.27 (dq, *J* = 23.4, 7.7 Hz, 7H), 4.28 (d, *J* = 5.9 Hz, 2H), 3.48 (s, 2H). ^13^C NMR (126 MHz, DMSO-*d*_6_) δ 170.62, 161.76, 151.45, 149.65, 141.95, 139.94, 136.90, 132.77, 129.67, 128.74, 127.97, 127.69, 127.23, 122.24, 121.04, 42.71, 42.32. LC-MS (ESI), RT = 1.360 min; *m/z* 380.2 [M+H]^+^. HRMS (ESI), RT= 5.732 min, *m/z* 380.11667 [M+H]^+^, formula C_21_H_18_ClN_3_O_2_.

##### 4.1.2.15 N-(4-(2-(benzylamino)-2-oxoethyl)phenyl)-5-chloronicotinamide (4o)

The compound was synthesized by reacting 5-chlornicotinic acid with **3a** according to the procedure for the synthesis of **4h**. The reaction produced 0.381 g (62%). Melting point 231°C. ^1^H NMR (400 MHz, DMSO-*d*_6_) δ 10.48 (s, 1H), 9.05 (s, 1H), 8.83 (d, *J* = 2.4 Hz, 1H), 8.53 (t, *J* = 5.9 Hz, 1H), 8.42 (s, 1H), 7.69 (d, *J* = 8.1 Hz, 2H), 7.29 (t, *J* = 7.2 Hz, 4H), 7.24 (d, *J* = 7.3 Hz, 3H), 4.28 (d, *J* = 5.9 Hz, 2H), 3.47 (s, 2H); ^13^C NMR (126 MHz, DMSO-*d*_6_) δ 170.61, 162.87, 151.01, 147.57, 139.94, 137.42, 135.43, 132.75, 132.27, 131.45, 129.75, 128.74, 127.70, 127.23, 120.80, 42.71, 42.30. LC-MS (ESI), RT = 1.252 min; *m/z* 380.2 [M+H]^+^. HRMS (ESI), RT= 5.821 min, *m/z* 380.11641 [M+H]^+^, formula C_21_H_18_ClN_3_O_2_.

##### 4.1.2.16 N-(4-(2-(benzylamino)-2-oxoethyl)phenyl)-5-chlorofuran-2-carboxamide (4p)

The compound was synthesized by reacting 5-chlorofuran-2-carboxylic acid with **3a** according to the procedure for the synthesis of **4h**. The reaction produced 0.375 g (75%). Melting point 217°C. ^1^H NMR (400 MHz, DMSO-*d*_6_) δ 10.19 (s, 1H), 8.52 (t, *J* = 6.0 Hz, 1H), 7.64 (d, *J* = 8.1 Hz, 2H), 7.41 (d, *J* = 3.6 Hz, 1H), 7.31 (t, *J* = 7.4 Hz, 2H), 7.28 – 7.16 (m, 5H), 6.74 (d, *J* = 3.7 Hz, 1H), 4.27 (d, *J* = 5.9 Hz, 2H), 3.46 (s, 2H). ^13^C NMR (126 MHz, DMSO-*d*_6_) δ 170.63, 155.47, 147.57, 139.94, 138.26, 137.08, 132.48, 129.69, 128.73, 127.69, 127.22, 120.90, 117.34, 109.97, 42.70, 42.28. LC-MS (ESI), RT = 1.299 min; *m/z* 369.2 [M+H]^+^. HRMS (ESI), RT= 6.036 min, *m/z* 369.10063 [M+H]^+^, formula C_20_H_17_ClN_2_O_3_.

##### 4.1.2.17 3-Chloro-N-(4-(2-oxo-2-(phenethylamino)ethyl)phenyl)benzamide (4q)

This compound was prepared according by reacting **3b** with 3-chlorobenzoyl chloride according to the procedure described for the synthesis of **4a.** The compound was white solid (69%). Melting point 160°C. ^1^H NMR (850 MHz, DMSO-*d*_6_) δ 10.32 (s, 1H), 8.09 (s, 1H), 8.01 (t, *J* = 1.82 Hz, 1H), 7.92 (dd, *J* = 1.56, 7.78 Hz, 1H), 7.89 - 7.91 (m, 1H), 7.66 - 7.69 (m, 2H), 7.57 (t, *J* = 7.79 Hz, 1H), 7.27 - 7.30 (m, 2H), 7.19 - 7.22 (m, 2H), 7.17 - 7.19 (m, 2H), 3.37 (s, 2H), 3.27 - 3.30 (m, 2H), 2.71 (t, *J* = 7.27 Hz, 2H). ^13^C NMR (214 MHz, DMSO-*d*_6_) δ 170.6, 166.5, 164.4, 139.9, 137.6, 137.4, 133.7, 133.2, 132.5, 131.8, 131.2, 130.9, 129.6, 129.3, 129.2, 128.8, 128.4, 127.9, 126.9, 126.5, 120.8, 42.4, 40.8, 35.5. LC-MS (ESI), RT = 2.4 min; *m/z* 394.1 [M+H]^+^.

##### 4.1.2.18 3-Chloro-N-(4-(2-((furan-2-ylmethyl)amino)-2-oxoethyl)phenyl)benzamide (4r)

This compound was prepared according by reacting **3c** with 3-chlorobenzoyl chloride according to the procedure described for the synthesis of **4a**. The compound was white solid (42%). Melting point 206°C. ^1^H NMR (850 MHz, DMSO-*d*_6_) δ 10.32 (s, 1H), 8.51 (s, 1H), 8.01 (t, *J* = 1.82 Hz, 1H), 7.89 - 7.94 (m, 1H), 7.66 - 7.70 (m, 2H), 7.54 - 7.59 (m, 2H), 7.25 (d, *J* = 8.30 Hz, 2H), 6.38 - 6.40 (m, 1H), 6.21 (d, *J* = 3.11 Hz, 1H), 4.27 (d, *J* = 5.71 Hz, 2H), 3.43 (s, 2H). ^13^C NMR (214 MHz, DMSO-*d*_6_) δ 170.5, 166.5, 164.4, 152.7, 142.6, 137.7, 137.4, 133.7, 133.2, 132.3, 131.8, 131.2, 130.9, 129.7, 129.3, 128.4, 127.8, 126.9, 120.9, 110.9, 107.3, 42.1, 36.1. LC-MS (ESI), RT = 2.1 min; *m/z* 369.1 [M+H]^+^.

##### 4.1.2.19 N-(6-(2-(benzylamino)-2-oxoethyl)pyridin-3-yl)-3-chlorobenzamide (4s)

The compound was synthesized by reacting 3-chlorobenzoic acid and 2-(5-aminopyridin-2-yl)-*N*-benzylacetamide according to the procedure for the synthesis of **4h**. The reaction produced 0.407 g of the compound (51.6%). Melting point 183°C. ^1^H NMR (400 MHz, DMSO-*d*_6_) δ 10.51 (s, 1H), 8.84 (s, 1H), 8.59 (s, 1H), 8.12 (d, *J* = 8.4 Hz, 1H), 8.04 (s, 1H), 7.94 (d, *J* = 7.8 Hz, 1H), 7.64 (dd, *J* = 36.6, 7.8 Hz, 2H), 7.49 – 7.04 (m, 6H), 4.31 (s, 2H), 3.68 (s, 2H). ^13^C NMR (126 MHz, CDCl_3_) δ 169.72, 164.76, 152.00, 141.78, 139.91, 136.79, 134.30, 133.75, 132.12, 130.95, 128.72, 128.52, 127.92, 127.70, 127.21, 127.02, 123.95, 44.81, 42.73. LC-MS (ESI), RT = 1.247 min; *m/z* 380.2 [M+H]^+^. HRMS (ESI), RT= 5.72 min, *m/z* 380.11641 [M+H]^+^, formula C_21_H_18_ClN_3_O_2_.

### 4.2 Methods for Biological Experiments on Solid Tumor Cell Lines

All cell lines were purchased from commercial cell line banks. All biological work involving human cell lines and animal derived material were approved by the institutional ethical committee at the Center of Excellence in Genomic Medicine Research (Approval code: 13-CEGMR-Bioeth-2021). All experiments were performed in triplicate and IC_50_ values were calculated considering all results.

#### 4.2.1 Cell Culture

MCF7 (breast) or N87 (stomach) cancer cells were grown in Roswell Park Memorial Institute-1640 (RPMI-1640) medium (Thermo Fisher Scientific, Inc; Waltham, MA, USA), supplemented with 10% heat inactivated fetal bovine serum (FBS), 50 units/mL of penicillin and 50 mg/mL of streptomycin and maintained at 37°C in a humidified atmosphere containing 5% CO_2_. The cells were maintained as “monolayer culture” by serial subculturing.

#### 4.2.2 SRB Cytotoxicity Assay

Cytotoxicity was determined using Sulforhodamine B (SRB) method as previously described by Skehan et al. (66). Exponentially growing MCF7 and N87 cells were collected using 0.25% Trypsin-EDTA and were then seeded in 96-well plates at a density of 1000-2000 cells/well in 10% FBS and penicillin/streptomycin supplemented RPMI-1640 medium. After 24 h, cells were treated with different concentrations of test compounds and incubated for 72 h at 37°C in a humidified atmosphere containing 5% CO_2_. Next, the cells were fixed by adding trichloroacetic acid (TCA, 10% final conc. in wells) directly to the wells and incubated for 1 h at 4°C, then cells were washed with distilled water 5 times to remove excess TCA, media, serum proteins, and metabolites, followed by air drying. After that, the fixed cells were stained with 0.4% SRB dissolved in 1% acetic acid for 10 min at room temperature, and excess stain was removed by quickly rinsing with 1% acetic acid. The residual acetic acid was removed completely by air drying the plates for 24 h, and the dye was solubilized with Tris buffer (pH= 7.4, 10 mM) for 5 min on a shaker at 1600 rpm. The optical density (OD) of each well was measured spectrophotometrically at 564 nm with an ELISA microplate reader (ChroMate-4300, FL, USA). The IC_50_ values (concentration of drug resulting in 50% reduction in cell survival) were calculated according to the equation for Boltzman sigmoidal concentration–response curve using the nonlinear regression fitting models (Graph Pad, Prism Version 5).

#### 4.2.3 MTT Anti-Proliferative Assay

The test compounds were evaluated for anti-proliferative activity using the MTT viability assay against HCT116 cell line. Cells were seeded in triplicate in 96-well plates at a density of 10x 10^3^ cells/mL in a total volume of 200 µL per well. 0.1% of DMSO was used as a vehicle control. Each well was treated with 2 µL test compounds, which had been pre-prepared as stock solutions in ethanol to furnish the concentration range of study, 0.1 µM to 50 µM, and re-incubated for a further 72 h at 37°C in a humidified atmosphere containing 5% CO_2_. The culture medium was then removed, and the cells were washed with 100 µL PBS and 100 µL MTT was added, to reach a final concentration of 1 mg/mL MTT. Cells were incubated for 3 h in darkness at 37°C. After that, MTT solution was removed and MTT was solubilized by adding 200 mL DMSO. The cells were maintained at room temperature in darkness for 20 min to ensure thorough color diffusion before reading the absorbance using a microplate reader at 570 nm. All experiments were conducted in triplicates. Results were expressed as percentage viability relative to vehicle control (100%). Dose response curves were plotted and IC_50_ values (concentration of drug resulting in 50% reduction in cell survival) were obtained using the commercial software package Prism (GraphPad Software, Inc., La Jolla, CA, USA).

### 4.3 Methods for Biological Experiments on Leukemia Cell Lines

#### 4.3.1 Cell Culture and Reagents

HL60, K562, MV4-11 and NB4 were purchased from CLS Cell Line Service GmbH (Eppelheim, Germany). All cell lines were cultured in RPMI-1640 medium supplemented with 10% fetal bovine serum (FBS) (Thermo Fisher Scientific) and 0.1% ciprofloxacin (2.5mg/ml; Cipla Limited; Mumbai, India), and were maintained at 37°C in the presence of 21% O_2_ and 5% CO_2_.

#### 4.3.2 Cell Viability Assay

Cell viability was determined using the CellTiter^®^-Blue Cell Viability Assay kit (Promega, Madison, WI, USA). Approximately 10^4^ cells were counted and plated in a 96-well plate in the presence of test compounds ranging from 0.01 to 10 or 20 µM in triplicate and were incubated for 48 h at 37°C in a humidified incubator. After the incubation period was complete, 20 µL of CellTiter^®^-Blue Cell Viability Assay reagent was added to each well and incubated for additional 2 h for the development of florescence. Florescence was measured at Ex/Em 540/590 on SpectraMax^®^ i3 Multi-Mode microplate reader (Molecular Devices, LLC; San Jose, CA, USA) and plotted against drug concentration to determine the IC_50_ of the test compounds. The IC_50_ was obtained by non-linear regression model using GraphPad Prism 6.07 (GraphPad Software, Inc, USA).

#### 4.3.3 Cell Cycle Analysis

HL60 cells (3.5 x 10^5^) were seeded in a 6-well plate, were treated with appropriate concentrations of the test compound after attachment, and were incubated at 37°C for 48 h. The treated cells were then collected and washed twice with ice-cold PBS (1X). The washed cells were fixed using 80% ice cold ethanol and stored at -20°C overnight. Fixed cells were washed twice with PBS before staining with 10 µg of Hoechst 33342 (10 µg/mL; Thermo Fisher Scientific, Inc.) and incubated in the dark for 30 min on ice. Cells were washed again with PBS wash and a minimum total of 20,000 events were collected and analyzed using a BD FACSAria III flow cytometer (BD Biosciences, USA). Flowlogic software version 7.0/7.2.1 (Inivai Technologies Pvt Ltd, Victoria, Australia) was used to obtain the percentage distribution of cells in G1, S, and G2/M phases in the singlet-gated population. For validation purpose, we included KX2-391 as a positive reference in this assay. In agreement with literature, the IC_50_ values of this drug were 12.1 nM, 14.5 nM, 4.19 nM and 3.3 nM against HL60, K562, MV411 and NB4, respectively.

#### 4.3.4 Apoptosis Detection Analysis

Total apoptosis was detected using Annexin V-FITC Apoptosis Detection kit (Cell Signaling Technology, Danvers, MA, USA) as per the protocol. Briefly, 1.5 x 10^5^ of HL60 cells were plated in a 6-well plate with appropriate compound concentrations at 37°C for 48 h. After the incubation completed, cells were washed twice with ice-cold PBS (1X). Subsequently cells were stained with Annexin V-FITC/PI and incubated in the dark on ice for 20 min. After washing with PBS wash, the cells were analyzed by acquiring a minimum total of 5000 events using BD FACSAria III flow cytometer (BD Biosciences, USA).

#### 4.3.5 Tubulin Polymerization Assay

The assembly of purified bovine tubulin was monitored using the Tubulin Polymerization Assay kit, BK006 (Cytoskeleton Inc., Denver, CO, USA). The assay was carried out in accordance with the manufacturer’s instructions in the assay kit manual using the standard assay conditions. The values reported represent the average values from two independent assays. Purified (> 99%) bovine brain tubulin (3 mg/mL) in a buffer consisting of 80 mM PIPES (pH 6.9), 0.5 mM EGTA, 2 mM MgCl_2_, 1 mM GTP and 10% glycerol, was incubated at 37°C in the presence of either vehicle (2% v/v DMSO) or test compound (10 µM in DMSO). KX2-391 and Paclitaxel were used as positive controls (reference drugs). Light is scattered proportionally to the concentration of polymerized microtubules in the assay. Therefore, tubulin assembly was monitored turbidimetrically at 340 nm at 37°C in a Spectramax 340 PC spectrophotometer (Molecular Devices, Sunnyvale, CA, USA). The absorbance was measured at 30 s intervals for 60 min.

#### 4.3.6 Rhodamine 6G Efflux Assay

Approximately 1.5 x 10^5^ of K562_Adr_ cells were incubated with 0.5 µM of R6G in the presence of appropriate test compounds concentrations for 1 h at 37°C. The cells were washed with ice-cold PBS (1X) and incubated with the compounds without R6G for another 1 h. Subsequently cells were washed again and analyzed on BD FACSAria III flow cytometer (BD Biosciences, USA) for R6G florescence.

#### 4.3.7 PamChip^®^ Tyrosine Kinase Microarray

The compound **4e** was spiked into HL60 cell lysate at different concentrations (0.001, 0.01, 0.1, 1, 10 and 50 μM). In addition, DMSO was also spiked in HL60 lysate at low concentration (2% v/v) as control. Samples were profiled on PamGene’s Tyrosine Kinase (PTK) arrays in technical replicates, covering a total of 196 phosphorylation sites. Frozen HL60 cultured cell pellet was sent to PamGene. At PamGene, cells were lysed using a lysis buffer and proteases inhibitors cocktail. Protein concentration was measured using standard Bradford assay as per PamGene SOPs. These lysates were further used for generating the PTK activity profiles. For spiking, **4e** was received as powder form and dissolved in DMSO at PamGene facility followed by storing at -20°C in small aliquots. PamGene used its in-house bioinformatics toolbox in combination with other methods to generate hypotheses of signaling pathways.

#### 4.3.8 Human Phosphokinase Array Assay

The phosphorylation profile of 48 kinases was performed using the Proteome Profiler Human Phospho-Kinase Antibody Array Kit by R&D Systems (Minneapolis, MN, USA) according to the protocol. Briefly, the total cellular protein was extracted using the Lysis buffer and was quantified using Bio-Rad DC Protein Assay kit (Hercules, CA, USA). Nitrocellulose membrane set (A&B), pre-spotted with kinase-specific capture antibodies, were first blocked with Array Buffer-1 at RT for 30 min on a rocking platform shaker. Approximately 600 µg of protein concentrate was diluted in the buffer provided and was added onto the membrane set, and the membranes were incubated overnight at 4°C in an 8-well plate. Subsequently, membranes were washed with Wash Buffer and incubated with appropriate Detection Antibody cocktails for 2 h at RT. Streptavadin-HRP (1:2000) was added onto membranes and incubated for 30 min at RT. Membranes were washed, and Chemiluminescent Detection Reagents 1&2 were applied in equal volumes for detection. The signals were detected on C-DiGit^®^Blot Scanner (LI-COR, Odyssey Imaging Systems, Lincoln, Nebraska, USA) using Image Studio 5.0 software.

### 4.4 Physico-Chemical Property Assay

#### 4.4.1 Maximum Kinetic Aqueous Solubility

Solubility assays were performed using Millipore MultiScreen^®^ HTS-PFC Filter Plates designed for solubility assays (EMD Millipore, Billerica, MA). Assays were run in triplicate. The 96-well plates consist of two chambers separated by a filter. Liquid handling was performed using JANUS^®^ Verispan and MTD workstations (Perkin Elmer, Waltham, MA). 4 µL of **4e** or **4n** solution (10 mM in DMSO) was added to 196 µL of phosphate buffer (45 mM potassium phosphate, 45 mM sodium acetate, 45 mM ethanolamine, pH = 7.4) in the top chamber to give a final DMSO concentration of 2% and a theoretical drug concentration of 200 µM. Plates were gently shaken for 90 min and then subjected to vacuum. Insoluble drug is captured on the filter. 160 µL of the filtrate is transferred to 96-well Griener UV Star^®^ analysis plates (Sigma–Aldrich, St. Louis, MO) containing 40 µL of acetonitrile. The test compound concentration in the filtrate is measured by UV absorbance on a Spectromax^®^ Plus microplate reader (Molecular Devices, Sunnyvale, CA) using Softmax Pro software v.5.4.5. Absorbances at 5 wavelengths (280, 300, 320, 340, and 360 nm) were summed to generate the UV signal. Standard curves were generated by adding 4 µL of five concentrations of test compound in DMSO to 40 µL of acetonitrile in UV Star plates followed by 156 µL of the appropriate solubility medium. Analysis and statistics were performed using GraphPad^®^ Prism v.5.04. Data were reported as the maximum concentration observed in the filtrate.

#### 4.4.2 Stability in Mouse and Human Liver Microsomes

The clearance of **4e** and **4n** in mouse or human liver microsomes was determined at 37°C as previously described (67). Both mouse and human liver microsomes were obtained from Merck KGaA, (Darmstadt, Germany), Cat. No. M9441 (Mouse liver microsomes) and M0317 (Human liver microsomes). Assays were conducted in triplicate in 96-deep well polypropylene plates. The compounds were incubated with pooled liver microsomes from male CD-1 mice (Life Technologies, Grand Island, NY), tetra-sodium NADPH and magnesium chloride for 60 min at 37°C with gentle shaking. At five time points, reaction mixture aliquots were transferred to 96-shallow well stop plates on ice containing acetonitrile with 0.1 µM propafenone. Control reactions (lacking NADPH) were performed in a similar manner to demonstrate NADPH dependency of compound loss and assess the potential for hydrolysis of compounds in liver tissue. Standard curves for test compound were generated using 5 concentrations in triplicate that were processed as above but with zero incubation time. Stop plates were centrifuged at 2000 g for 10 min and then supernatant aliquots were transferred to a Waters Aquity^®^ UPLC 700 µL 96-well sample plate with cap mat (Waters, Milford, MA). The amount of compound remaining in the supernatant was quantified by LC/MS/MS using a Waters Xevo TQ MS (electrospray positive mode) coupled to a Waters Aquity^®^ UPLC (BEH column, C18). Propafenone was used as the internal standard. GraphPad^®^ Prism v 5.04 was used for nonlinear fitting of time course data to generate t_1/2_ values.

#### 4.4.3 Inhibition Assay of Human CYP450 Enzymes

The two compounds **4e** and **4n** were assessed for their ability to inhibit the three major human CYP450 enzymes, 3A4, 2D6 and 2C9. Assays were run in triplicate. Expressed enzymes in insect supersomes (Fisher Scientific, Waltham, MA) were used to minimize non-specific binding and membrane partitioning issues (68). The 3A4 assay uses testosterone as a substrate and analysis was performed by LC/MS/MS on a Waters Xevo TQ instrument as described above using positive electrospray ionization. Assay acceptance criteria was 20% for all standards and 25% for the LLOQ. The 2D6 and 2C9 assays use fluorescent substrates (3-[2-(*N*,*N*-diethylamino)ethyl]-7-methoxy-4-methylcoumarin for 2D6; 7-methoxy-4-(trifluoromethyl)coumarin for 2C9) and were analyzed on an Envision plate reader. GraphPad^®^ Prism v 5.04 was used for nonlinear fitting of data to generate IC_50_ values.

#### 4.4.4 Stability in Mouse Plasma

The assay was conducted at 37°C in triplicate. Test compounds or procaine (positive control) were tested at a final concentration of 1 µM in either 2.5% DMSO/CD-1 mouse plasma (Innovative Research, Novi, MI; sodium heparin added as anticoagulant; pH adjusted to 7.4 with 2N HCl on day of use) or 2.5% DMSO/PBS (pH 7.4: 136.9 mM NaCl, 2.68 mM KCl, 8.1 mM Na_2_HPO_4_, 1.47 mM KH_2_PO_4_, 0.9 mM CaCl_2_, 0.49 mM MgCl_2_). At seven time points, reaction mixture aliquots were transferred to 96-shallow well stop plates on ice containing acetonitrile with 0.1 µM propafenone. Samples were analyzed by LC/MS/MS on a Waters Xevo TQ instrument as described above using positive electrospray ionization. Assay acceptance criteria was 20% for all standards and 25% for the LLOQ. GraphPad^®^ Prism v. 5.04 was used for nonlinear fitting of data to generate t_1/2_ values.

#### 4.4.5 Binding to Mouse Plasma Proteins

The equilibrium dialysis method for determining plasma protein binding was performed as previously described using 96-well dialyzer plates with molecular weight cutoff of 5K (Harvard Aparatus, Holliston, MA) and a dual-plate rotator set to maximum speed (Harvard Aparatus, Holliston, MA) located in a 37°C incubator with a 10% CO_2_-atmospheric environment. Assays were run in triplicate. The test compounds were added to CD-1 mouse plasma (Innovative Research, Novi, MI, sodium heparin added as anticoagulant; pH adjusted to 7.4 with 2N HCl on day of use) in DMSO (final DMSO concentration 0.4%) to give 10 µM final concentration. Drug/plasma mixture and buffer (Dulbecco’s phosphate-buffered saline 1X without calcium and magnesium, Mediatech, Inc., Herndon, VA) were placed in their respective sides, wells were capped, and the plate was placed in the rotator and allowed to dialyze for 22 h. Following dialysis, aliquots of buffer and plasma mixture were removed and mixed with aliquots of the opposite matrix in 96-well deep plates. Concentrations of analytes from each side of the dialysis plate were determined by LC/MS/MS on a Waters Xevo TQ instrument as described above using positive electrospray ionization. Assay acceptance criteria was 20% for all standards and 25% for the LLOQ. The fraction unbound was calculated by dividing the drug concentration in the buffer side of the dialysis plate by the drug concentration in the plasma side.

## Data Availability Statement

The datasets presented in this study can be found in online repositories. The names of the repository/repositories and accession number(s) can be found in the article/**Supplementary Material**.

## Author Contributions

Conceptualization and design, ME-A and AO. Methodology, AM, AO, YM, SI, ME-A, FA, DS, and WC. Software, YM, ME-A, FA, and WC. Validation, ME-A, FA, and MKhan. Formal analysis, YM, MKhay, MKhan, DS, and ME-A. Investigation, AM, AO, MKhay, ME-A, FA, DS, MKhan, and WC. Resources, AM, MKhay, ME-A, and FA. Data curation, ME-A, FA, MKhan, SI, WC, and DS. Writing—original draft preparation, AM, YM, ME-A, FA, and MKhan. Writing—review and editing, WC, ME-A, and YM. Visualization, AM, YM, ME-A, FA, MKhan, and SI. Supervision, AO, MKhay, and ME-A. Project administration, ME-A and AO. Funding acquisition, MKhay. All authors have read and agreed to the published version of the manuscript.

## Funding

This research was funded by the Deanship of Scientific Research (DSR), King Abdulaziz University, Jeddah, the Kingdom of Saudi Arabia, under grant number RG-1436-166-537.

## Conflict of Interest

The authors declare that the research was conducted in the absence of any commercial or financial relationships that could be construed as a potential conflict of interest.

## Publisher’s Note

All claims expressed in this article are solely those of the authors and do not necessarily represent those of their affiliated organizations, or those of the publisher, the editors and the reviewers. Any product that may be evaluated in this article, or claim that may be made by its manufacturer, is not guaranteed or endorsed by the publisher.

## Abbreviations

ABCB1, ATP Binding Cassette B1; ADME, absorption/distribution/metabolism/elimination; AKT; AML, acute myeloid leukemia; BCR–ABL, breakpoint cluster region protein- abelson; Chk-2, checkpoint kinase 2; CML, chronic myeloid leukemia; CREB, cAMP response element binding protein; CYP 450, cytochrome P450; Eph, erythropoietin-producing human hepatocellular receptor; ERK1/2, extracellular signal-related kinase 1/2; ETK/BMX, ETK tyrosine kinase, also known as Bone Marrow tyrosine kinase gene on chromosome X; FLT3-ITD, FMS-like tyrosine kinase 3-internal tandem duplication; GSK-3α/β, glycogen synthase kinase 3α/β; HSP27, heat shock protein 27 kDa; IGF-1R, insulin-like growth factor 1 receptor; ITK, IL-2-inducible T-cell kinase; JNK, c-Jun NH2-terminal kinase; c-KIT, KIT receptor kinase; LFC, log fold change; MAPK, Mitogen-activated protein kinase; mTOR, mammalian target of rapamycin; NADPH, nicotinamide adenine dinucleotide phosphate; PBS, phosphate-buffered saline; PDGFR, platelet-derived growth factor receptor; PDK1, phosphoinositide-dependent kinase-1; PI3K, phosphatidylinositol 3-kinase; PK/PD, pharmacokinetic and pharmacodynamic; SAR, structure-activity relationship; SFK, Src-family kinases; STK, serine-threonine kinase; TEC, Tec protein tyrosine kinase; TXK, TXK tyrosine kinase; YKI, tyrosine kinase inhibitor.
