# Supplementary material for: SAR Probing of KX2-391 Provided Analogues With Juxtaposed Activity Profile Against Major Oncogenic Kinases
Source: Front Oncol. 2022 May 20;12:879457. doi: 10.3389/fonc.2022.879457 (PMC9166630; doi:10.3389/fonc.2022.879457)
Supplement: Supplementary file 1 [file DataSheet_1.docx]

Supplementary Material

# S1. Spectra of Screened Compounds

## 2-(4-Aminophenyl)-*N*-benzylacetamide (3a)

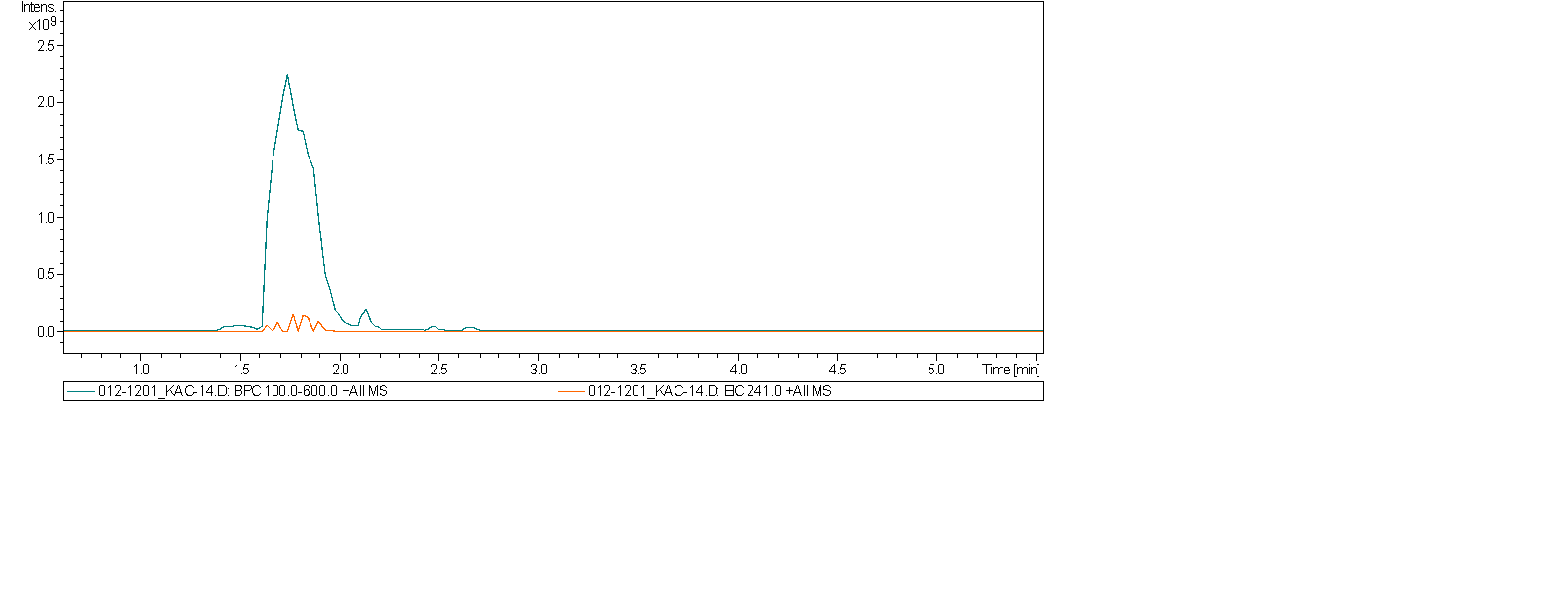


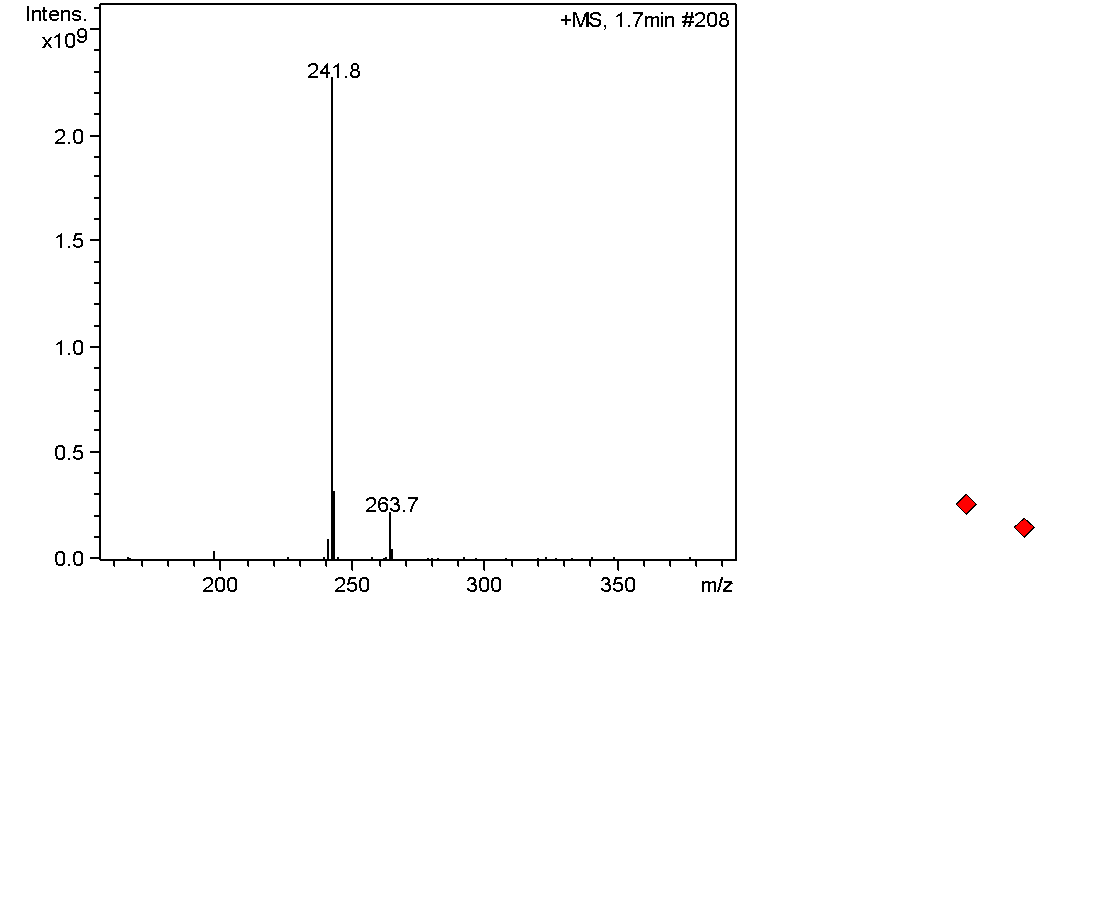


## 2-(4-acetamidophenyl)-*N*-benzylacetamide (4a)

## *N*-(4-(2-(benzylamino)-2-oxoethyl)phenyl)propionamide (4b)

## *N*-(4-(2-(benzylamino)-2-oxoethyl)phenyl)benzamide (4c)

## *N*-(4-(2-(benzylamino)-2-oxoethyl)phenyl)-3-fluorobenzamide (4d)

## *N*-(4-(2-(benzylamino)-2-oxoethyl)phenyl)-3-chlorobenzamide (4e)

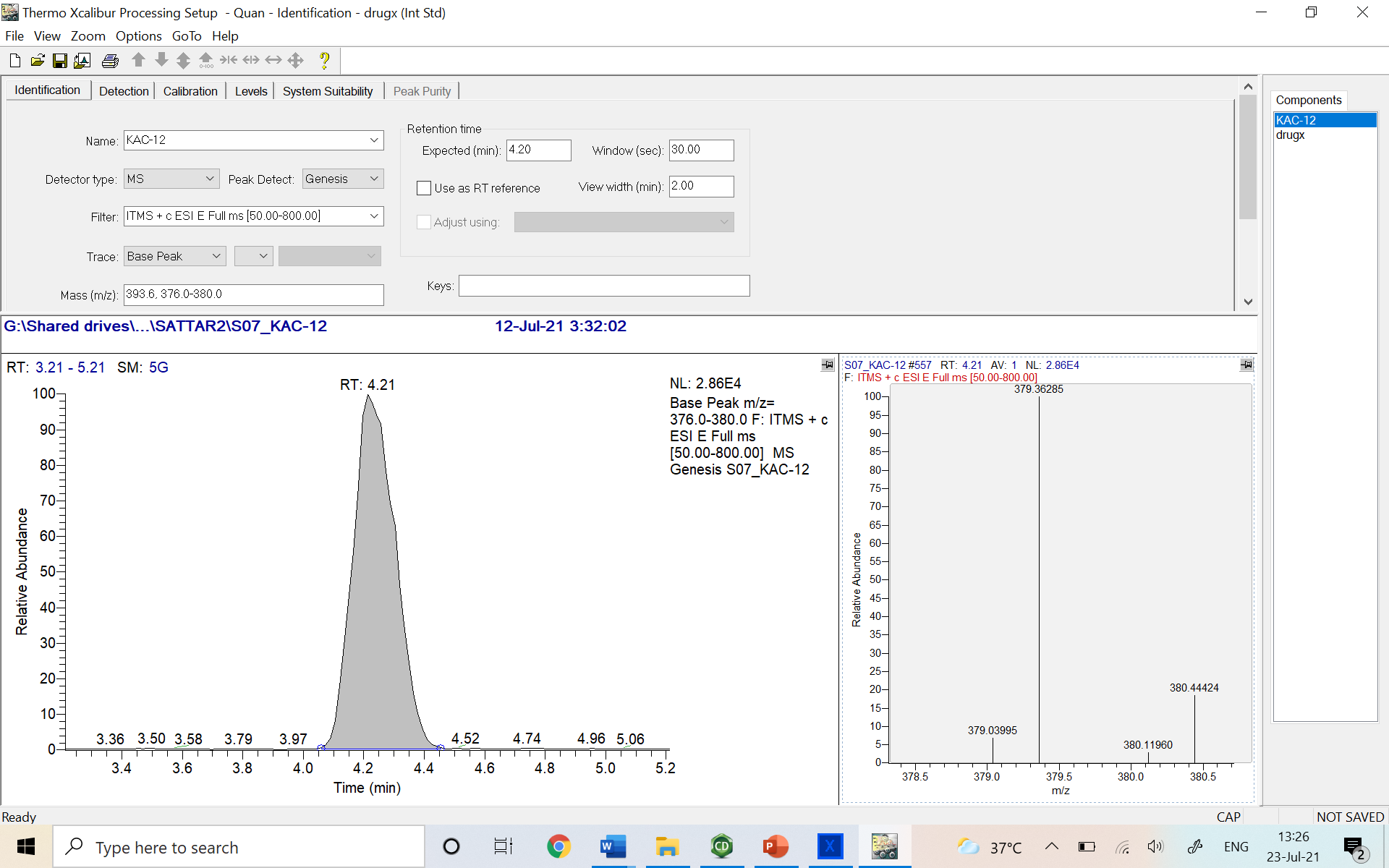


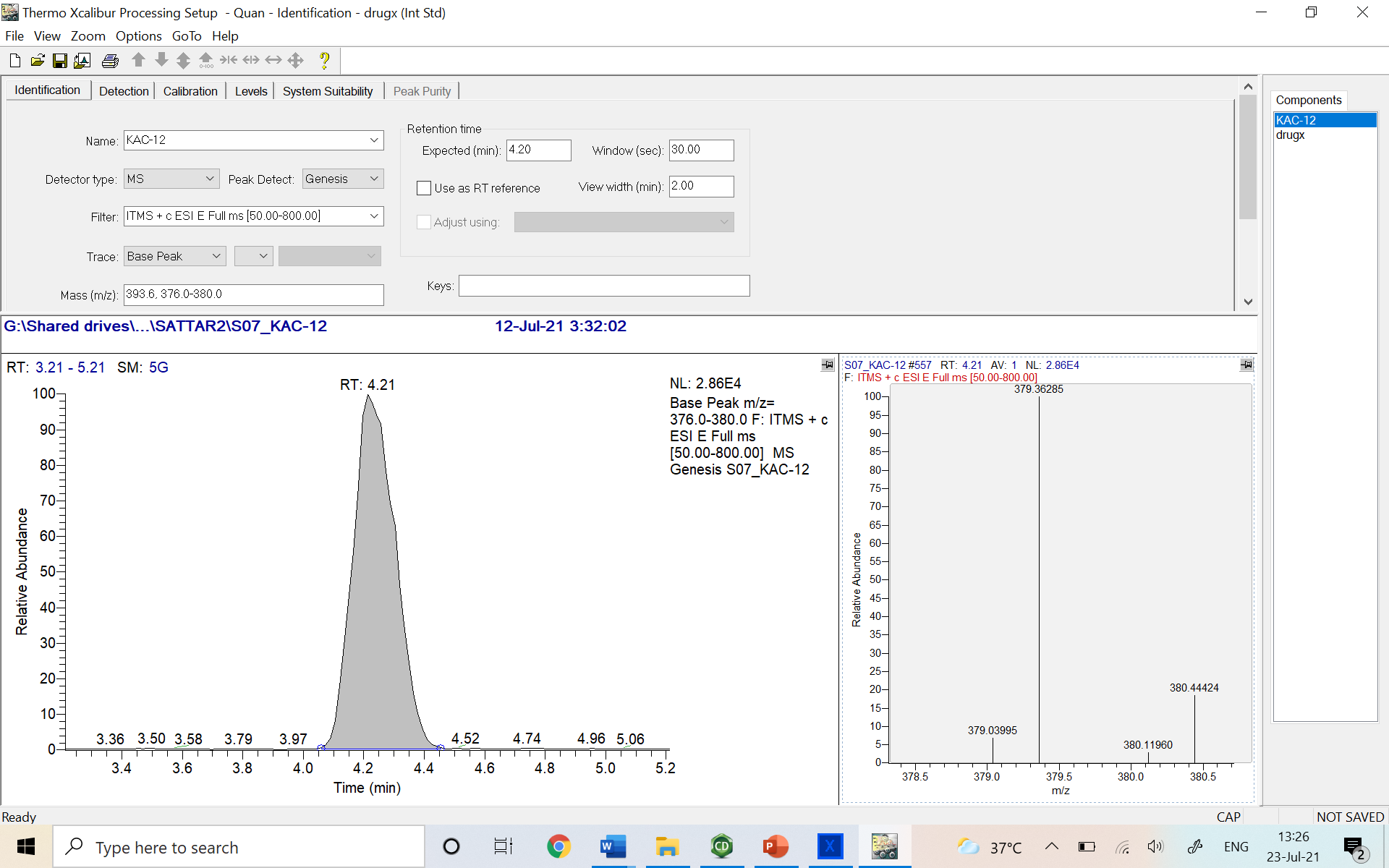


## *N*-(4-(2-(benzylamino)-2-oxoethyl)phenyl)-4-chlorobenzamide (4f)

## *N*-benzyl-2-(4-((4-methylphenyl)sulfonamido)phenyl)acetamide (4g)

## *N*-(4-(2-(benzylamino)-2-oxoethyl)phenyl)-3-methoxybenzamide (4h)

## *N*-(4-(2-(benzylamino)-2-oxoethyl)phenyl)-3-chloro-4-(2-morpholinoethoxy) benzamide (4i)

## *N*-(4-(2-(benzylamino)-2-oxoethyl)phenyl)-3-chloro-4-methoxybenzamide (4j)

## *N*-(4-(2-(benzylamino)-2-oxoethyl)phenyl)-3,4-dimethoxybenzamide (4k)

## *N*-(4-(2-(benzylamino)-2-oxoethyl)phenyl)-3-chloro-4-(2-morpholinoethoxy) benzamide (4l)

## *N*-(4-(2-(benzylamino)-2-oxoethyl)phenyl)-2-chloroisonicotinamide (4m)

## *N*-(4-(2-(benzylamino)-2-oxoethyl)phenyl)-6-chloropicolinamide (4n)

## *N*-(4-(2-(benzylamino)-2-oxoethyl)phenyl)-5-chloronicotinamide (4o)

## *N*-(4-(2-(benzylamino)-2-oxoethyl)phenyl)-5-chlorofuran-2-carboxamide (4p)

## 3-Chloro-*N*-(4-(2-oxo-2-(phenethylamino)ethyl)phenyl)benzamide (4q)

## 3-Chloro-*N*-(4-(2-((furan-2-ylmethyl)amino)-2-oxoethyl)phenyl)benzamide (4r)

## *N*-(6-(2-(benzylamino)-2-oxoethyl)pyridin-3-yl)-3-chlorobenzamide (4s)

**Table S1: List of kinases in the Proteome Profiler Human Phospho-Kinase Array Kit (**[link](https://www.rndsystems.com/products/proteome-profiler-human-phospho-kinase-array-kit_ary003c?utm_source=biocompare&utm_medium=referral&utm_campaign=product_ARY003C&utm_term=proteomeprofilerantibodyarrays&utm_content=editorial)**)**

| Akt 1/2/3 (S473) | HSP60 | PRAS40 (T246) |
| --- | --- | --- |
| Akt 1/2/3 (T308) | JNK 1/2/3 (T183/Y185, T221/Y223) | Pyk2 (Y402) |
| beta-Catenin | Lck (Y394) | RSK1/2 (S221/S227) |
| Chk-2 (T68) | Lyn (Y397) | RSK1/2/3 (S380/S386/S377) |
| c-Jun (S63) | MSK1/2 (S376/S360) | Src (Y419) |
| CREB (S133) | p38 alpha (T180/Y182) | STAT1 (Y701) |
| EGF R (Y1086) | p53 (S15) | STAT2 (Y689) |
| eNOS (S1177) | p53 (S392) | STAT3 (S727) |
| ERK1/2 (T202/Y204, T185/Y187) | p53 (S46) | STAT3 (Y705) |
| Fgr (Y412) | P70 S6 Kinase (T389) | STAT5a/b (Y699) |
| GSK-3 alpha/beta (S21/S9) | p70 S6 Kinase (T421/S424) | STAT6 (Y641) |
| GSK-3 beta (S9) | PDGF R beta (Y751) | WNK-1 (T60) |
| HSP27 (S78/S82) | PLC gamma-1 (Y783) | Yes (Y426) |
